# Application of Artificial Intelligence Methods for Predicting the Compressive Strength of Self-Compacting Concrete with Class F Fly Ash

**DOI:** 10.3390/ma15124191

**Published:** 2022-06-13

**Authors:** Miljan Kovačević, Silva Lozančić, Emmanuel Karlo Nyarko, Marijana Hadzima-Nyarko

**Affiliations:** 1 Faculty of Technical Sciences, University of Pristina, Knjaza Milosa 7, 38220 Kosovska Mitrovica, Serbia; 2 Faculty of Civil Engineering and Architecture Osijek, Josip Juraj Strossmayer University of Osijek, Vladimira Preloga 3, 31000 Osijek, Croatia; mhadzima@gfos.hr; 3 Faculty of Electrical Engineering, Computer Science and Information Technology, Josip Juraj Strossmayer University of Osijek, Kneza Trpimira 2B, 31000 Osijek, Croatia; karlo.nyarko@ferit.hr

**Keywords:** self-compacting concrete, Class F fly ash, compressive strength, machine learning, artificial neural networks, regression trees, Gaussian process regression

## Abstract

Replacing a specified quantity of cement with Class F fly ash contributes to sustainable development and reducing the greenhouse effect. In order to use Class F fly ash in self-compacting concrete (SCC), a prediction model that will give a satisfactory accuracy value for the compressive strength of such concrete is required. This paper considers a number of machine learning models created on a dataset of 327 experimentally tested samples in order to create an optimal predictive model. The set of input variables for all models consists of seven input variables, among which six are constituent components of SCC, and the seventh model variable represents the age of the sample. Models based on regression trees (RTs), Gaussian process regression (GPR), support vector regression (SVR) and artificial neural networks (ANNs) are considered. The accuracy of individual models and ensemble models are analyzed. The research shows that the model with the highest accuracy is an ensemble of ANNs. This accuracy expressed through the mean absolute error (MAE) and correlation coefficient (R) criteria is 4.37 MPa and 0.96, respectively. This paper also compares the accuracy of individual prediction models and determines their accuracy. Compared to theindividual ANN model, the more transparent multi-gene genetic programming (MGPP) model and the individual regression tree (RT) model have comparable or better prediction accuracy. The accuracy of the MGGP and RT models expressed through the MAE and R criteria is 5.70 MPa and 0.93, and 6.64 MPa and 0.89, respectively.

## 1. Introduction

The significant production of Portland cement results in the emission of large amounts of carbon dioxide, and the replacement of cement with fly ash is one way to reduce the emission of greenhouse gases and thus contribute to sustainable development. ASTM C618 defines two classes of fly ash used in concrete: (1) Class F with low calcium content; and (2) Class C with high calcium content. Class F is usually obtained by burning anthracite or bituminous coal, and Class C is usually obtained by burning lignite or partially bituminous coal [1,2]. The ASTM C618 standard defines the physical, chemical, and mechanical properties of this class of fly ash.

The content of Portland cement is usually such that it has 65% lime, part of which becomes free and available during hydration, and its mixing with Class F fly ash as pozzolanic material forms new binders while improving many properties of the resulting concrete. The advantages of using fly ash in concrete are increased strength, increased workability, reduction in the appearance of excess water during installation and lower water demand, lower water permeability and reduced penetration of chloride ions, reduced heat of hydration, greater resistance to sulfates, greater resistance to alkaline reactivity and reduced shrinkage during drying [3]. 

SCC is a high-performance concrete, the main characteristic of which is its fluid and viscous consistency allowing it to flow through densely reinforced structural elements without the addition of outside energy for compaction [4,5,6]. The use of ordinary Portland cement concrete in the filling of more complex formwork requires mechanical vibration.

This unique property of SCC can be achieved by using complementary cementitious materials such as Class F fly ash. Fly ash particles behave as one type of miniature ball bearings inside the concrete mix, which gives them the effect of lubricant, which is a particularly useful property in SCC [3].

The application of fly ash in concrete results in a slightly slower reaction and less heat per unit time than Portland cement, which reacts more quickly. Less heat release is especially important in massive concrete structures, which is an advantage of using fly ash in such structures.

In the problem of predicting the compressive strength (CS) of SCC in recent years, we have an increasing application of machine learning (ML) methods and algorithms (Table 1). The most common is the application of ANN models. Siddique et al. [7] worked on a model of the application of ANN for predicting the CS of SCC at different sample ages from 7 to 365 days. MAE, RMSE, and correlation coefficient criteria ^®^ were used as accuracy criteria. In their paper, Asteris et al. [8] investigated the application of ANN in predicting the CS of SCC with the addition of fly ash after 28 days. The basis for the development of the model was tests of 169 samples collected from the published literature. Douma et al. [9] investigated the application of ANN to model the properties of SCC with the addition of fly ash. The prediction of fresh concrete properties and CS after 28 days was analyzed. Models with a total of 6 constituents as input variables were analyzed, and the base consisted of 114 examined samples. Models were evaluated through MSE, coefficient of determination, and MAPE criteria. The research recommended an ANN model with one hidden layer of 17 neurons as optimal. Asteris and Kolovos [10] worked on the application of a surrogate model to assess the CS of SCC after 28 days. Eleven different constituents of SCC were considered, one of which was fly ash. Different architectures of ANNs trained on a set of data from 205 examined samples were analyzed, and their accuracy was evaluated through the correlation coefficient R. They recommended using ANN as an optimal model. Saha et al. [11] researched the application of the support vector machines (SVM) model with different kernel functions in predicting the properties of fresh and hardened concrete with the addition of fly ash. The models were evaluated via a correlation coefficient. A model with an exponential radial basis function (ERBF) was recommended as the optimal model. Research related to the examination of the properties of fresh and hardened high-volume fly ash concrete was conducted by Azimi-Pour and others [12]. The application of SVM with different linear and nonlinear kernel functions was tested, based on the tested samples in a fresh and hardened state. The determination coefficient, RMSE, and MAPE were used as accuracy criteria. A model with an RBF kernel function was recommended as a model with higher accuracy compared to others. In their study of CS prediction modeling lightweight self-compacting concrete with the addition of fly ash, Zhang et al. [13] analyzed hybrid procedures in which they combined the beetle antennae search algorithm (BAS) with the random forest (RF) algorithm. The BAS algorithm was used to optimize the hyperparameters of the RF model. Song et al. [14] worked on the application of regression trees (RT), ANNs, genetic engineering programming, and boosting regressor models in the development of models for predicting the CS of SCC. The accuracy of the model was assessed using cross-validation, and the criteria used were the coefficient of determination ( $R^{2}$ ), root mean error (RME), and root mean squared error (RMSE). Research recommended the use of ensemble algorithms in terms of accuracy. Hadzima-Nyarko et al. [15] investigated the application of SCC with the use of rubber and fly ash additives. The paper analyzed the application of different GPR models. This study showed that Gaussian process regression (GPR) modeling is an appropriate method for predicting the CS of SCC with recycled tire rubber particles and fly ash. Their results were further confirmed by scanning electron microscopy (SEM) images. Kovacevic et al. [16] conducted a similar study to create a model for predicting the CS of lightweight concrete with the addition of rubber and fly ash. The research concluded that GPR models are the optimal choice in this case. The combination of ANN models where network parameters are optimized using the firefly optimization algorithm in the prediction of CS samples of different ages was considered by Pazouki et al. [17]. Farooq et al. [18] performed research on determining a suitable model for predicting the CS of concrete modified with fly ash after 28 days. Models of ANNs, support vector machines, and gene expression programming (GEP) models were tested. The GEP model was proposed as the optimal model. In their study, de-Prado-Gil et al. dealt with the application of the ensemble methods: random forest (RF), K-nearest neighbor (KNN), extremely randomized trees (ERT), extreme gradient boosting (XGB), gradient boosting (GB), light gradient boosting machine (LGBM), category boosting (CB) and the generalized additive models (GAMs), and for the development of the models, 515 samples were collected. The results indicated that the RF models have a strong potential to predict the CS of SCC with recycled aggregates [19].

The novelty of this research is the use of a significant number of state-of-the-art ML methods trained on a significant set of experimental data. This paper is also important as it defines the optimal model for predicting the CS of SCC of different sample ages. The research describes in detail the procedure for optimizing the hyperparameters of the model and gives a detailed comparative analysis of all models. MGGP models, models based on regression trees (RT, bagged, RF, and boosted trees), the application of SVM models with linear and nonlinear kernel functions (linear, RBF, and sigmoid), different GPR models, ANN models, and ensembles composed of individual models of ANNs. This paper also investigates the application of GPR methods with automatic relevance determination (ARD) covariance functions, which, according to the authors, has not been used to date for modeling the CS of SCC with Class F fly ash.

## 2. Methods

Machine learning (ML) is a branch of artificial intelligence and includes methods of training algorithms such that they can learn from data and make decisions and predictions, which can be applied in the modeling of the behavior of structures and materials (Table 1). The strength of ML methods lies in the fact that these methods can represent a general relationship or function directly from experimental data to model the behavior of complex systems with multiple influence variables, whose effects, both individual and synergistic, are unknown or difficult to predict. In addition, these methods can process a large amount of data that contain, not only, complex information about the observed phenomenon but also “noise”, which is an integral part of experimental data. The application of ML methods has become more pronounced in recent years due to the increasing amount of data available as well as due to significant progress in the field of computing.

### 2.1. Multi-Gene Genetic Programming (MGGP)

MGGP is an ML method whose root is in biological processes that it seeks to emulate. It can be used to define symbolic expressions to predict a continuous output variable for some input variables (symbolic regression). This method generates appropriate expressions (represented by trees) and these expressions are improved in iterations using evolutionary methods.

In contrast to traditional regression analysis methods where there is bias in modeling, the structure is assumed in advance, and the parameters of the model are determined from the data; in MGGP, however, the empirical data themselves define the structure of the model. Unlike other machine learning methods such as ANN, the MGGP method provides insight into the physical process under consideration [20]. MGGP enables the creation of a number of models that are defined by different symbolic expressions and that have different accuracy and complexity.

**Figure 1:**
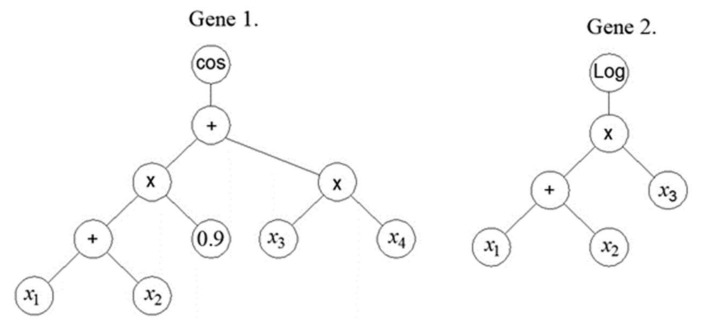
An example of an MGGP model that has two genes.

In the first generation, the MGGP method generates a random population of models. An individual model in a population is represented by one or more individual trees or one or more individual genes (Figure 1). Each tree represents one model gene that is generated using the appropriate mathematical functions (plus, minus, times, …). using M corresponding input variables $x_{1},x_{2},\ldots ,x_{M}$ and ephemeral random constants (ERCs) generated within the appropriate ranges. Tree nodes end in either the appropriate input variables or constants, and are called terminal nodes, while other nodes are called functional nodes.

If the number of genes within an individual is denoted by $N_{G}^{k}$ , then that individual can be written as $J_{k}=\{G_{1,k,}G_{2,k,}$ …, $G_{i,k,}$ ,…, $\;G_{N_{G}^{k},k,}$ } where $G_{i,k}$ represents the ith gene within the kth individual.

Each model is limited in terms of the maximum number of individual trees that make it up, which is denoted by $N_{mg}$ and in terms of the depth of the trees generated. Models do not have to be composed of the same number of genes, and individuals within the first generation have the greatest diversity.

**Figure 2:**
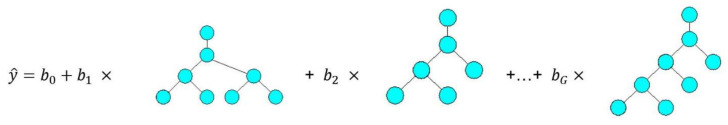
The general structure of the MGGP model.

The general structure of MGGP models is illustrated in Figure 2. The MGGP regression model can also be considered as a pseudo linear model in the sense that it represents a linear combination of the nonlinear transformations of input variables.

The individuals that make up the next generation are created using crossover, mutation, and the direct copying of the individuals of the previous generation. In the crossover, the pre-selection of individuals is necessary. In the crossover, either the entire gene can be replaced or only a partial gene can be replaced. Two-point high-level crossover operation allows the replacement of entire genes between two parent individuals by defining two random points of intersection. Consider this in the following example.

Let individual $J_{1}$ contain the following genes [ $G_{1,1\;}G_{2,1\;}G_{3,1}\;G_{4,1\;}]$ and let individual $J_{2}$ contain the following genes $\left[G_{1,2\;}\;G_{2,2\;}\;G_{3,2}\;G_{4,2\;}\;G_{5,2\;}\right]$ . Let us denote by <> the genes that are randomly included with two cross-sections in both models, i.e.,

$$J_{1}:\;[G_{1,1\;}<G_{2,1\;}G_{3,1}>G_{4,1\;}]\;,$$

$$J_{2}:\;[G_{1,2\;}\;G_{2,2\;}<G_{3,2}>G_{4,2\;}\;G_{5,2\;}].$$

Parts of the model or whole genes covered by random cross-sections are marked in bold and they are exchanged in offspring $O_{1\;}$ and $O_{2}.\;$ As a result of this crossover, the number of genes will decrease in one offspring, while the number of genes will increase in the other offspring [20].

$$O_{1}:\;[G_{1,1\;}\;G_{3,2}\;G_{4,1\;}]\;,$$

$$O_{2}:\;[G_{1,2\;}\;G_{2,2\;}\;G_{2,1\;}G_{3,1}\;G_{4,2\;}\;G_{5,2\;}].$$

**Figure 3:**
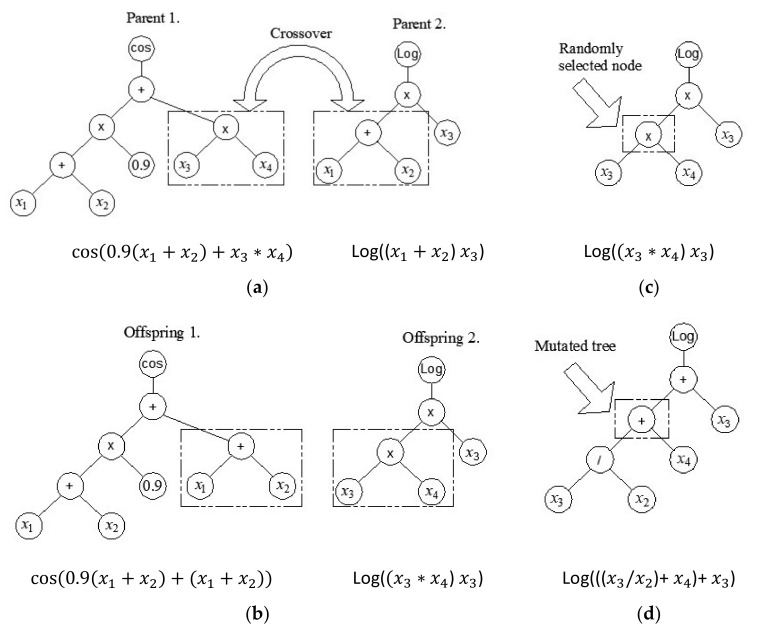
Crossover and mutation operation in MGGP: (**a**) random selection of parent tree nodes; (**b**) exchange of parents’ genetic material; (**c**) random node selection in tree mutation; and (**d**) mutation of a randomly selected part of a tree.

Crossover is also implemented at the level of one gene (low-level crossover) where the structure of a part of the gene changes (Figure 3a,b). However, only part of the gene may be exchanged (that is, only part of the tree is exchanged). In addition to crossover, mutation at the level of a single gene is also possible (Figure 3c,d). In a mutation, one gene and one node within it are selected at random, and then the appropriate mutation is applied, where a randomly created subtree is added at the site of the selected node.

The prediction of the output y for the training data is given by [20]:

(1) $$\hat{y}=b_{0}+b_{1}t_{1}+b_{2}t_{2}+\ldots +b_{G}t_{G}$$

where $b_{0}$ is the bias term, $b_{i}$ is the ith scaling parameter, $t_{i}$ is the ( N×1) vector of outputs from the ith tree (gene). 

G is defined as $N\times \left(G+1\right)\;$ gene response matrix i.e., $G=[\;1\;t_{1}$ $t_{2}$ … $t_{G}$ ], and b as $\left(G+1\right)\times 1)$ vector of the coefficients $b_{0}$ , $b_{1}$ , …, $b_{G}$ i.e., $b=\left[b_{0}\;b_{1}\;\;b_{2}\ldots \;b_{G}\right]$ . In matrix G , the first column is composed of ones and dimensionality N×1 to include bias input [20].

Hence, the previous Equation (1) can be written as:

(2) $$\hat{y}=Gb$$

The least-squares estimate of the vector b can be computed from the training data as [20]:

(3) $$b={\left(G^{T}G\right)}^{-1}G^{T}y$$

The RMSE is then calculated and used as the fitness (objective) function to be minimized within the MGGP algorithm. Here, we can see how the MGGP model is linear in terms of parameters and the coefficients are determined by the method using the least squares method.

In each succeeding generation, individuals are selected for breeding using regular tournament selection that uses only RMSE or Pareto tournament or a mixture of both. In Pareto tournament selection, each individual is selected probabilistically based on its RMSE (fitness) value and its expressional complexity. Expressional complexity is computed by summing together the node count itself within stable and all possible subtrees that can be obtained from that stable [20]. Thus, if there are two trees with the same number of nodes, flatter trees will have an advantage over deep trees in terms of expressional complexity values. As there are several trees (genes) in the MGGP model, the expressional complexity of the model is equal to the sum of the expressive complexity of all trees (genes) in the model.

### 2.2. Regression Tree Ensembles

Methods based on classification trees can be applied to both regression and classification problems. A tree is built from the root to the leaves, the so-called greedy approach. In the beginning, all instances of space belong to the same set, after which space is successively divided into subsets. It is necessary to determine the variable and the value of the split point corresponds to the minimum value of the expression (4) obtained by analyzing all input variables in the model and it serves as the point at which the division of space will be performed [21,22,23].

(4) $$\underset{j,\;s}{min}\left[\underset{c_{1}}{min}\underset{x_{i}\in R_{1}\left(j,\;s\right)}{\sum }{\left(y_{i}-c_{1}\right)}^{2}+\underset{c_{2}}{min}\underset{x_{i}\in R_{2}\left(j,\;s\right)}{\sum }{\left(y_{i}-c_{2}\right)}^{2}\right]$$

where ${\hat{c}}_{1}$ = $\;ave\left(y_{i\;}|x_{i}\in R_{1}\left(j,\;s\right)\right)$ and ${\hat{c}}_{2}$ = $\;ave\left(y_{i\;}|x_{i}\in R_{2}\left(j,\;s\right)\right).$

After finding the split variable j and the best split point s , the procedure is continued by further splitting these areas until a certain stop criterion is met. This approach is said to be greedy because at each step the best division is determined on the basis of the state in the observed step, i.e., it does not consider how the division that takes place will perform in the next steps and which division could result in better ones in the following steps.

**Figure 4:**
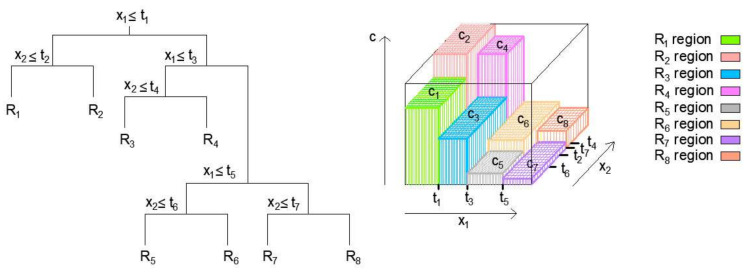
Example of the segmentation of variable spaces into regions and 3D regression surfaces for the created regression tree [16].

After the segmentation of space into M areas $R_{1},R_{2},\ldots ,\;R_{M}$ , the model output for a given test observation gives a value equal to the mean value of all observations of the region to which the test sample belongs (Figure 4).

When an individual tree model has poor generalization indicators on the test set, the problem can be overcome by using a larger number of regression trees by using ensemble techniques such as bagging, random forest, and boosting. 

With bagging algorithms, it is necessary to apply the method of bootstrap sampling or sampling with replacement, so that a training set of the same size as the original is formed. The procedure needs to be repeated several times or as many times as there are individual models within the ensemble, so that each set of data generated in this way is used to create one model of the regression tree [21,22,23].

The process of forming regression trees in the RF algorithm is based on the bootstrap method similar to the bagging algorithm [24]. However, when regression trees are formed, only a certain subset of variables is randomly selected, instead of using all available variables or the total number of variables. Splits in the model are only performed on these variables. If p is the total number of input variables or predictors in the training data, the algorithm will narrow the selection to m=p / 3 variables when selecting a new branching variable [21,25]. For each branching, a new random sample of variables is taken into consideration.

**Figure 5:**
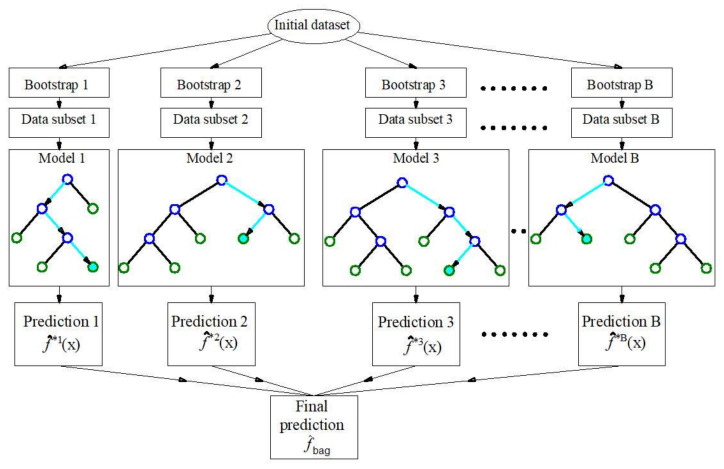
Bootstrap aggregation (bagging) in regression tree ensembles [25].

By averaging all B individual models (for bagging and RF algorithm), a model is obtained whose predictive function at point x is determined (Figure 5) by the following expression:

(5) $$\hat{f}\left(x\right)=\frac{1}{B}\overset{B}{\underset{b=1}{\sum }}\;{\hat{f}}^{*b}\left(x\right).$$

**Figure 6:**
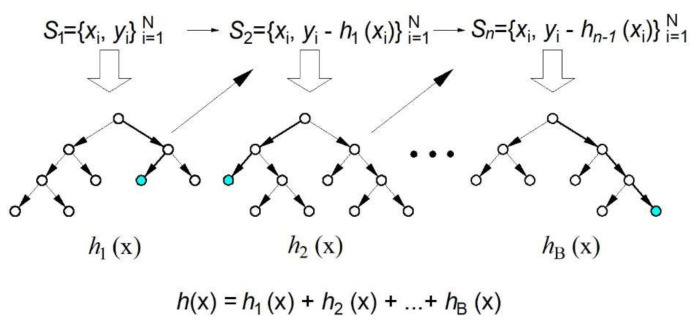
Gradient boosting in regression tree ensembles [25].

In simple aggregation for the bagging and RF algorithm, the models are constructed completely independently. The basic idea of gradient boosting is to build an ensemble by adding a model per model (Figure 6), where each of the models is trained to compensate as much as possible the weaknesses of the current set of models, i.e., to strengthen it. The basic idea comes from gradient optimization methods, which are based on fixing the current solution of the optimization problem by adding a vector proportional to the negative value of the gradient of the function being minimized [26,27,28]. This makes sense, since a negative gradient value indicates the direction of decreasing function. When the quadratic error function is applied, the model reinforcement is implemented by each subsequent model trying to approximate the residuals of previous models.

### 2.3. Support Vector Regression (SVR)

If $x_{i}$ denotes the i-th element of an *m*-dimensional vector representing the inputs to the model and *y_i_* denotes the corresponding answers to the values of the input vectors, their values make up the following dataset $\left\{(x_{1},\;y_{1}),\;(x_{2},\;y_{2}),\ldots ,\;(x_{n},\;y_{n}),\;\right\}\in \;R^{m\;}\times R$ .

**Figure 7:**
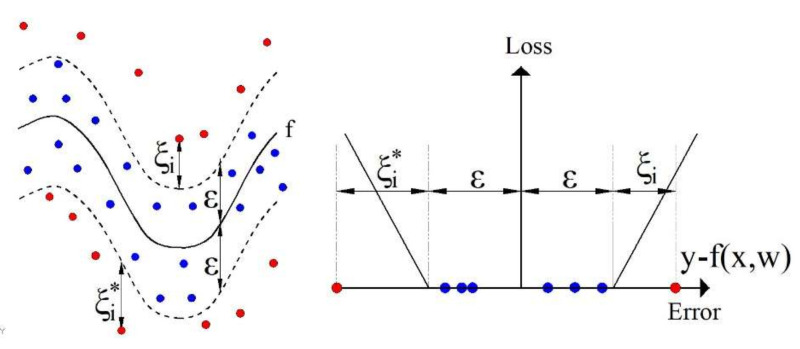
Nonlinear SVR with linear loss function with an ε-insensitivity zone.

The SVR algorithm can approximate an unknown function by tolerating errors within the ε value or the so-called ε tube, but at the same time, there is a possibility to take into account errors outside the ε tube by introducing linear loss function (Figure 7) defined by the following expression [29,30]:

(6) y−fx,wε=0       if         y−fx,w≤ε  y−fx,w−ε    otherwise.

For the case of a linear SVR model, the approximation function can be written as:

(7) $$f\left(x,w\right)=\langle \omega ,x\rangle +b,where\;\omega \in Χ,b\in R$$

where 〈ω,x〉 represents the scalar product.

The function of empirical risk that needs to be minimized is given by the following expression (8):

(8) $$R_{emp}^{\varepsilon }\left(w,b\right)=\frac{1}{l}\overset{n}{\underset{i=1}{\sum }}{\left|y_{i}-f\left(x,w\right)\right|}_{\varepsilon .}\;$$

The SVR algorithm minimizes the values of empirical risk $R_{emp}^{\varepsilon }$ and $\left||w^{2}|\right|$ at the same time. By minimizing vector w , predictions are less sensitive to perturbations in features, i.e., outliers, and on the other hand, important features are selected, putting small coefficients on those that do not contribute to the model. Taking into account the above, the problem is reduced to minimizing the following function:

(9) $$R_{emp}^{\varepsilon }\left(w,b\right)=\frac{1}{l}\overset{n}{\underset{i=1}{\sum }}{\left|y_{i}-f\left(x,w\right)\right|}_{\varepsilon .}\;$$

(10) $$\mathrm{subject}\;\mathrm{to}\;\left\{\begin{array}{c} \;y_{i}-\langle \omega ,x_{i}\rangle -b\leq \varepsilon +\xi_{i}\\ \langle \omega ,x_{i}\rangle +b-y_{i}\leq \varepsilon +\xi_{i}^{*}\\ \xi_{i},\xi_{i}^{*}\geq 0 \end{array}\right.$$

The constant C  > 0 represents a parameter of the model that punishes errors greater than ε and $\xi_{i}$ and $\xi_{i}^{*}$ are the slack variables, as shown in Figure 7.

By introducing the Lagrange function with a dual set of variables, this problem can be solved and the final solution can be written as:

(11) $$\omega =\overset{n}{\underset{i=1}{\sum }}\left(\alpha_{i}+\alpha_{i}^{*}\right)x_{i\;}and\;thus\;f\left(x\right)=\overset{n}{\underset{i=1}{\sum }}\left(\alpha_{i}+\alpha_{i}^{*}\right)\langle x_{i},x\rangle +b\;$$

where $\alpha_{i}$ , $\alpha_{i}{}^{*}$ , and b are the parameters obtained by minimizing the error function.

In the case when the nonlinear function is approximated, the so-called kernel trick is used, where it is necessary to map $x_{i}$ to a higher dimensional space using the mapping $\Phi :x\to \Phi \left(x\right)$ ; hence, the scalar product can be replaced with $k\langle x_{i},x\rangle =\langle \Phi (x_{i}),\Phi \left(x\right)\rangle$ . The final approximate function in this case becomes:

(12) $$f\left(x\right)=\overset{n}{\underset{i=1}{\sum }}\left(\alpha_{i}+\alpha_{i}^{*}\right)k\langle x_{i},x\rangle +b$$

The most commonly used kernel functions are linear (13), polynomial (14) and RBF (15), defined by the following expressions, respectively [31]:

(13) $$\langle kx_{i},x\rangle =\langle x_{i},x\rangle$$

(14) $$k\langle x_{i},x\rangle =exp\left(-\gamma ||x_{i}-x^{2}||\right),\;\gamma >0$$

(15) $$k\langle x_{i},x\rangle =\tanh\left(\gamma \langle x_{i},x\rangle +r\right),\;\gamma >0$$

### 2.4. Gaussian Process Regression (GPR)

This section introduces the GPR models which solve a regression task to find a function that returns a real value, denoted as f: ${\mathbb{R}}^{m}$ ⟼ ℝ for a given dataset in pairs $(x_{1},\;y_{1}$ ), $\left(x_{2},\;y_{2}\right)$ , …, $(x_{n},\;y_{n}$ ), where $x_{i}$ denotes the input vector and $\;y_{i}$ represents the scalar value of the output [32].

An input x can be viewed as a location where an unknown function f  that represents a random variable is approximated. In the general case, domain ℝ can be divided into three disjoint sets, training set X , test set $X_{*}$ , and the set of input values different from the training and test set $X_{0}$ :

(16) $$X=\left[\begin{array}{c} x_{1}\\ x_{2}\\ x_{3}\\ \ldots \\ x_{n} \end{array}\right],\;X_{*}=\left[\begin{array}{c} x_{*1}\\ x_{*2}\\ x_{*3}\\ \ldots \\ x_{*n_{*}} \end{array}\right],\;X_{0}=\left[\begin{array}{c} x_{01}\\ x_{02}\\ x_{03}\\ \ldots \\ x_{0n} \end{array}\right]$$

X is a vector of length n , $X_{*}$ is a vector of length $n_{*}$ and $X_{0}$ is a vector of infinite length $n_{0}$ .

When modeling the values of possible values of the unknown function f at some location X , it is assumed that it has the form of a Gaussian distribution function. A specific value of the function represents only the random value of the unknown function f with the corresponding probability. For each of the three previously mentioned disjoint sets of inputs, there are corresponding output values: $f\left(X\right)$ *,* a vector of random variables of length n , $f\left(X_{*}\right)$ , a vector of random variables of length $n_{*}$ , and $f\left(X_{0}\right)$ a vector of random variables of infinite length *nₒ*.

In GPR, a multivariate Gaussian distribution is used over the random variables $f\left(X\right)$ *,* $\;f\left(X_{*}\right)$ , and $f\left(X_{0}\right)$ , specified by a mean vector and a covariance matrix:


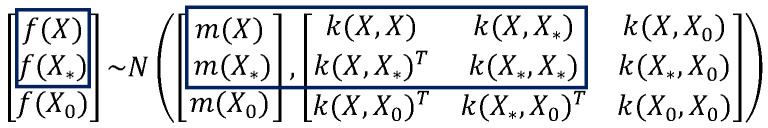
(17)


The mean function *m* gives the expected value of those random variables, the mean vector $m\left(X\right)$ for random variable $f\left(X\right)$ when x=X , the mean vector $m\left(X_{*}\right)$ for random variable $f\left(X_{*}\right)$ when $x=X_{*},$ and the mean vector $m\left(X_{0}\right)$ for random variable $f\left(X_{0}\right)$ when $x=X_{0}$ . In many modeling cases, satisfactory results are obtained by assuming that the mean function m is equal to zero (or equal to some constant value) because the original data can always be normalized to have a mean value of zero (or some constant value).

Additionally, it is necessary to define a covariance function *k* that occurs within the covariance matrix, which is called the kernel function. The kernel function *k* is used to determine the covariance between every two random values $f\left(x\right)$ and $f\;\left(x^{\prime }\right)$ and it represents one of the most important steps in the development of GPR models. One of the most commonly used kernels is defined by the expression:

(18) $$k\left(x,x^{\prime }\right)=\sigma^{2}exp\left(-\frac{{\left(x-\;x^{\prime }\right)}^{2}}{2l^{2}}\right)$$

in this expression, exp is the exponential function, l is called the lengthscale, and $\sigma^{2}$ is the signal variance. They are **model parameters**. 

Based on the adopted kernel, all elements of the covariance matrix can be determined. The matrix $k\;\left(X,\;X\right)\;$ is of dimensionality n×n , the matrix $k\left(X,X_{*}\right)$ is of dimensionality $n\times n_{*}$ , and the matrix $k\left(X_{*},X_{*}\right)$ is of dimensionality $n_{*}\times n_{*}$ .

The remaining five elements of the covariance matrix in equation (17) relating to $X_{0}$ ( $k\left(X,X_{0}\right),\;k\left(X_{*},X_{0}\right),k{\left(X,X_{0}\right)}^{T},k{\left(X_{*},X_{0}\right)}^{T},\;k\left(X_{0},X_{0}\right),\;$ have infinite dimensions with respect to infinite dimensionality $X_{0}$ , but due to the Gaussian marginalization rule, they can be omitted and only the distribution over the random variables $f\left(X\right)$ and $f\left(X_{*}\right)$ (parts from the joint probability density highlighted with blue boxes) can be considered. Joint probability density over the random variables $f\left(X\right)$ and $f\left(X_{*}\right)$ is called prior and it can also be written in the following form:

(19) $$p\left(\left[\begin{array}{c} f\left(X\right)\\ f\left(X_{*}\right) \end{array}\right]\right)=\frac{1}{{\left(2\pi \right)}^{\frac{n+n_{*}}{2}}det{\left(K\right)}^{\frac{1}{2}}}exp{\left(-\frac{1}{2}\left(\left[\begin{array}{c} f\left(X\right)\\ f\left(X_{*}\right) \end{array}\right]-\left[\begin{array}{c} m\left(X\right)\\ m\left(X_{*}\right) \end{array}\right]\right)\right)}^{T}K^{-1}\left(\left[\begin{array}{c} f\left(X\right)\\ f\left(X_{*}\right) \end{array}\right]-\left[\begin{array}{c} m\left(X\right)\\ m\left(X_{*}\right) \end{array}\right]\right)$$

Let us now introduce the relationship between the random variable $y\left(X\right)$ which represents the observations and the random variable $f\left(X\right)$ in the form of the following expression:

(20) $$p\left(y\left(X\right)\left|f\left(X\right)\right.\right)=N\left(y\left(X\right);f\left(X\right),\;\eta^{2}I_{n}\;\right),$$

where *η²* is called noise variance and $I_{n}$ is the identity matrix with size n×n .

The regression function $y\left(X\right)$ can be written using the Gaussian linear transformation form in the following way:

(21) $$y\left(X\right)=I_{n}f\left(X\right)+\varepsilon ,\;\mathrm{where}\;\varepsilon \sim N\left(0,\eta^{2}I_{n}\;\right).$$

Using the above formula and by applying the multivariate Gaussian marginalization rule to expression (17), the distribution of the random variable y (X ) can be obtained without using $f\left(X\right).$ By applying the Gaussian marginalization rule, the following expression is obtained:

(22) $$f\left(X\right)\sim N\left(m\left(X\right),\;k\left(X,X\right)\right),$$

and then, by applying the Gaussian linear transformation rule resulting in:

(23) $$y\left(X\right)\;)\sim N\left(m\left(X\right),\;K+\eta^{2}I_{n}\right),$$

Its probability density function is defined by the following expression (24) and is called marginal likelihood:

(24) $$p\left(y\left(X\right)\right)=\frac{1}{{\left(2\pi \right)}^{\frac{n}{2}}det{\left(\;K+\eta^{2}I_{n}\right)}^{\frac{1}{2}}}exp{\left(-\frac{1}{2}\left(y\left(X\right)-m\left(X\right)\right)\right)}^{T}{\left(\;K+\eta^{2}I_{n}\right)}^{-1}\left(y\left(X\right)-m\left(X\right)\right).$$

The marginal likelihood is a function of arguments: $y\left(X\right)$ and the model parameters $\left\{l,\;\;\sigma^{2},\;\eta^{2}\right\}$ . Determining the parameters of the model is done by maximizing this expression. If the expression for marginal likelihood is logarithmized, identical results are obtained by maximizing the previous expression for $p\left(y\left(X\right)\right)$ . For this reason, the determination of model parameters is performed by applying gradient procedures to the expression for log marginal probability.

In the next step, it is necessary to use the Bayesian method to calculate the posterior from the prior and the likelihood. To calculate the posterior $P\left(f\left(X_{*}\right)\left|y\left(X\right)\right.\right)$ , the fact that $f\left(X_{*}\right)$ and $y\left(X\right)$ are multivariate Gaussian random variables whose distribution is known is taken into account. The distribution for $f\left(X_{*}\right)$ is thus given by the following expression:

(25) $$f\left(X_{*}\right)\sim N(m\left(X_{*\;}\right),\;k\left(X_{*\;},X_{*\;}\right),$$

the expression for the common distribution $f\left(X_{*}\right)$ and $y\left(X\right)$ can be written as:

(26) $$p\left(\left[\begin{array}{c} f\left(X_{*}\right)\\ y\left(X\right) \end{array}\right]\right)=\sim \left[\;\begin{array}{c} m\left(X_{*\;}\right)\\ m\left(X\right) \end{array}\right],\left[\begin{array}{cc} k\left(X_{*},X_{*}\right) & k\left(X_{*\;},\;X\right)\\ k{\left(X_{*\;},\;X\right)}^{T} & k\left(X,X\right)+\eta^{2}I_{n} \end{array}\right].$$

After applying the conditional rule for multivariate Gaussian, a distribution for the posterior can be obtained:

(27) $$f\left(X_{*}\right)\left|y\left(X\right)\right.\sim N\left(\mu_{*},\sigma_{*}^{2}\right)$$

with the posterior mean and posterior covariance [32]:

(28) $$\mu_{*}=m\left(X_{*\;}\right)+k\left(X_{*\;},\;X\right){\left(k\left(X,X\right)+\eta^{2}I_{n}\right)}^{-1}\left(y\left(X\right)-m\left(X\right)\right)\;\sigma_{*}^{2}=k\left(X_{*},X_{*}\right)-k\left(X_{*\;},\;X\right){\left(k\left(X,X\right)+\eta^{2}I_{n}\right)}^{-1}k{\left(X_{*\;},\;X\right)}^{T}$$

The posterior for the observation random variable $y\left(X_{*}\right)\left|y\left(X\right)\right.$ is:

(29) $$y\left(X_{*}\right)\left|y\left(X\right)\right.=I_{\eta_{*}}(f\left(X_{*}\right)\left|y\left(X\right))+\epsilon_{*}\right.$$

where $\epsilon_{*}\sim N\left(0,\eta^{2}I_{n_{*}}\right)$ , $I_{n_{*}}$ is the identity matrix of size $n_{*}\times n_{*}$ .

By applying the multivariate Gaussian linear transformation rule, a distribution for $y\left(X_{*}\right)\left|y\left(X\right)\right.$ can be obtained:

(30) $$p\left(y\left(X_{*}\right)\left|y\left(X\right))=N(\right.I_{\eta_{*}}\mu_{*},\;\;\;\;I_{n_{*}}\eta_{*}^{2}I_{n_{*}}{}^{T}+\sigma^{2}I_{n_{*}}\right)=N(\mu_{*},\sigma_{*}^{2}+\eta^{2}I_{n_{*}})$$

### 2.5. Artificial Neural Networks (ANNs)

**Figure 8:**
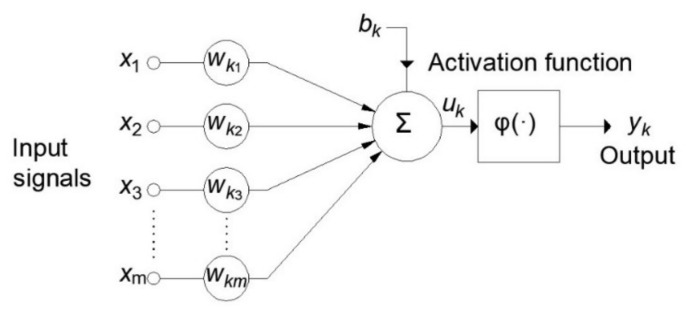
Nonlinear neuron model.

The ANN can be described as the mathematical representation of parallel information processing in a way similar to the human brain. The basic structural element (Figure 8) of ANNs is the artificial neuron. The artificial neuron model consists of the following elements:

1. A set of connections (synapses) where each connection has a certain weight;
2. A sum function where the input signals are collected;
3. Activation function, which limits the output of neurons.

Each of the input signals (inputs) $x_{j}$ is multiplied by the corresponding weight $w_{kj}$ before reaching the summation block, where the individual multiplied inputs are added together and a bias is added to them. The output from the sum function is the input to the activation function.

The output of the activation function is at the same time the output of neuron $y_{k}$ . In the neuron model shown in Figure 8, the bias $b_{k}$ is included which has the function of increasing or decreasing the input to the activation function. Mathematically, this can be described by the following equations:

(31) $$u_{k}=\overset{m}{\underset{j=0}{\sum }}w_{kj}x_{j,}\;y_{k}=\varphi \left(u_{k}\right).$$

where $x_{1},\;x_{2},\;.\;.\;.\;,\;x_{m}$ are input signals, and $w_{k1},\;w_{k2},\;.\;.\;.\;,\;w_{km}$ are the corresponding weights for neuron k , where its bias has an assigned value of $x_{0}=+1$ , and the corresponding weight is denoted by $w_{k0}=b_{k}.$

**Figure 9:**
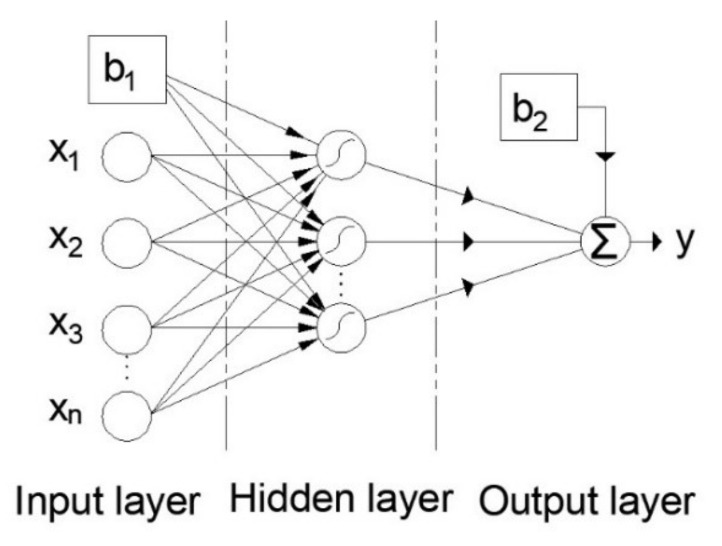
Multilayer perceptron artificial neural network (MLP-ANN).

One of the most commonly used ANN models is the so-called multilayer perceptron—MLP. An MLP is an ANN with forward signal propagation that consists of at least three layers: an input layer, hidden layer, and output layer. The general structure of the MLP model is given in Figure 9.

The optimal number of neurons in the hidden layer depends on many factors, including the number of inputs and outputs, the number of training pairs, the size of the noise in the training pairs, the complexity of the error function, network architecture, and training algorithm. One of the recommendations for adopting an upper limit for the number of hidden layer neurons $N_{H}$ is the following expression [16,25]:

(32) $$N_{H}\leq min\left(2N_{I}+1,\;\frac{N_{S}}{N_{I}+1}\;\right)\;$$

where $N_{I}$ is the number of input layer neurons and $N_{S}$ is the number of training samples.

The output provided by the network usually differs from the desired one, i.e., the ANN model gives an error that is a function of the adjustable network parameters, i.e., the weight between the network neurons. A variant of the backpropagation algorithm called the Levenberg-Marquardt (LM) algorithm was used to train the ANN model and its detailed description can be found in [33].

The accuracy of a single neural network model when a smaller set of data is available can be improved by training a larger number of ANNs and finding the average output value of such a so-called ensemble model. For each of the individual ANN models within the ensemble, an appropriate set of training data is required, which is formed by the Bootstrap method, where a set of an identical number of samples is formed from the original set by random selection with replacement.

The procedure is repeated until the appropriate number of ANN models within the ensemble is reached. The output of the ensemble represents the mean value of the output of the individual ANNs that form the ensemble.

## 3. Dataset

**Figure 10:**
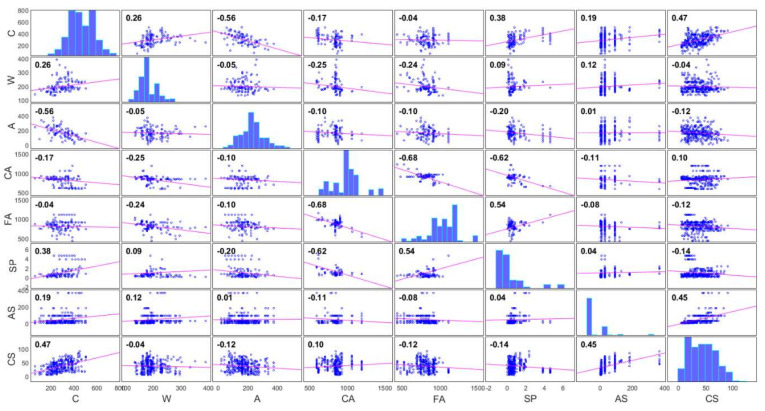
Correlation matrix of model variables.

In order to form a CS prediction model for different ages of SCC samples with the addition of Class F fly ash, it is necessary to form a sufficiently large set of test data of such concrete. In this case, a database of tests of SCC samples from the published literature was used [34,35,36,37,38,39,40,41,42,43,44,45,46,47,48,49,50,51,52,53,54,55]. The total number of tested samples used for modeling consisted of a total of 327 tested samples with different ages of the tested sample from 1 day to 365 days. Concrete constituents were analyzed as input variables: cement (C), water (W), Class F fly ash (A), coarse aggregate (CA), fine aggregate (FA), superplasticizer (SP), and the age of samples (AS). The output variable was the strength of such concrete at a cylinder pressure of 100 mm × 200 mm expressed in MPa. The complete database is available as Supplementary Materials (Supplementary Materials S1). The correlation matrix of model variables is given in Figure 10. 

Figure 10 indicates the correlation between model variables. Based on the value of the correlation coefficient, it can be seen that in addition to a certain correlation between compression strength as an output variable with some input variables (e.g., cement, sample age, etc.), there is an intercorrelation between the input variables (e.g., superplasticizer to fine aggregate ratio, the ratio of fly ash to cement, etc.). The application of machine learning methods is extremely effective in modeling just such problems with intercorrelation. In addition, histograms arranged along the main diagonal indicate the balance of the dataset. All diagrams are automatically created by applying the corrplot built-in function (Matlab 2020a) to the attached dataset.

When applying different machine learning algorithms in order to define prediction models, it is important to know the statistical indicators of the data used. The models that are being formed generalize within the data on which the model training was performed. The selection of the training and test dataset was implemented by first mixing the total dataset using random permutations of samples using the built-in **randperm** function of the Matlab program, and then randomly 80% of the samples for model training and 20% for model testing. This 80:20 division of data is a standard procedure in machine learning (Table 2). All analyzed models were trained on an identical training set, while the accuracy of the model was assessed using the criteria root mean square error (RMSE), mean absolute error (MAE), Pearson’s linear correlation coefficient ©, and mean absolute percentage error (MAPE) on the identical test set.

## 4. Results

In the MGGP method, different values of the number of genes and different depths of trees that determine the complexity of the model were analyzed. The procedure itself was iteratively repeated 10 times due to the random initiation of model parameters, and the obtained models were eventually merged into one final population. In the training procedure, the sum of RMSE values and the expression complexity of the model were taken into account as a function of error. Setting the parameters is given in Table 3.

**Figure 11:**
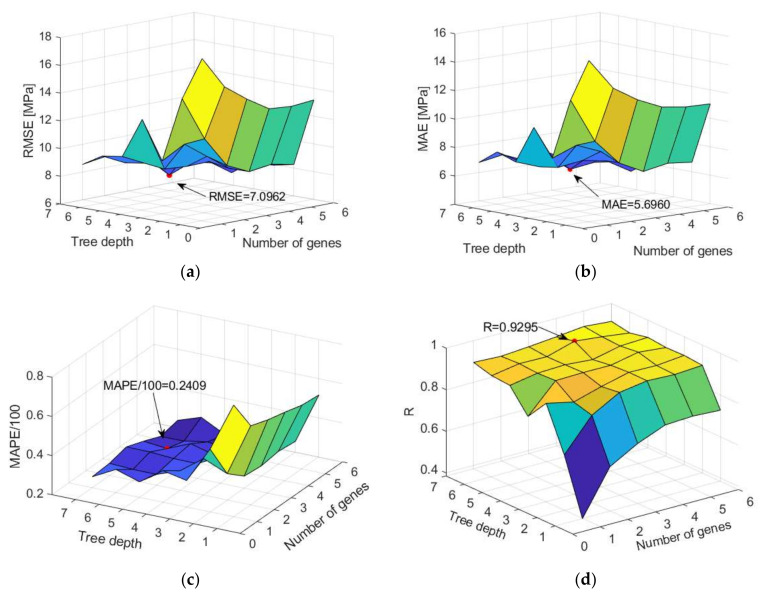
Comparison of different accuracy criteria for MGGP model as a function of gene number and tree depth (**a**) RMSE, (**b**) MAE, (**c**) MAPE, and (**d**) R.

Models with 1–6 genes and the maximum tree depth varying from 1 to 7 were analyzed. A model with four genes and maximum tree depth limited to six models was obtained with the highest accuracy on the test dataset (Figure 11).

**Figure 12:**
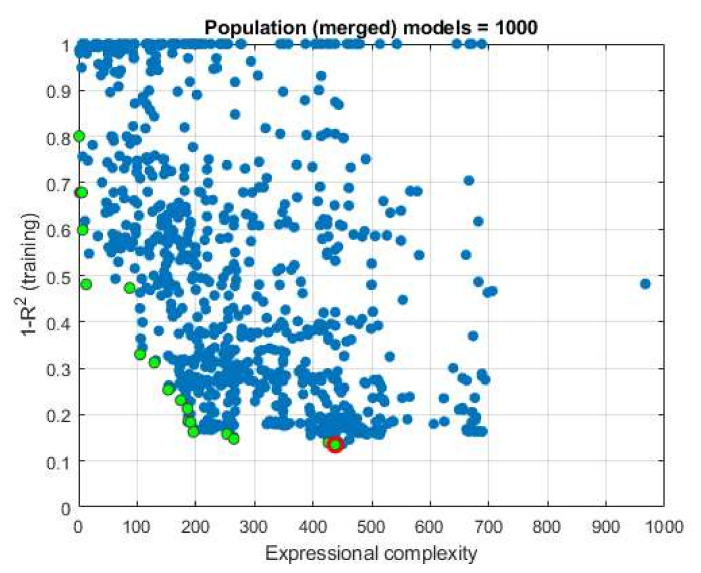
Display of models that make up the Pareto front marked with green circles, while the optimal model (Model ID 269) is marked with a red circle.

In this particular case, as a result of ten implemented iterations over 1000 generations, a definite set of 1000 potential models was obtained. From the obtained models, models were analyzed that stand out in terms of the correlation coefficient and in terms of expression complexity and form the so-called Pareto front (Figure 12).

From the models that make up the Pareto front (Figure A2), five models can further be distinguished in terms of accuracy expressed by the coefficients $R^{2}$ whose values are 0.837, 0.842, 0.852, 0.860, and 0.865. These models are further analyzed because of their similar accuracy and the significant difference in the complexity of the expressions themselves, due to the separation of the most accurate and simplest expression. The accuracy of these models in terms of defined criteria is given in the Table 4. The analysis showed that, in this case, the most complex model (Model ID 269) is also the most accurate model.

The analytical expression of the obtained optimal model for concrete strength (CS) consists of four terms and a member representing bias:

$$\sigma =b+\sigma_{1}+\sigma_{2}+\sigma_{3}+\sigma_{4}=74+{\;\sigma }_{1}+\sigma_{2}+\sigma_{3}+\sigma_{4}$$

$$\sigma_{1}=0.0001\left(1.24A-2.47C+1.24\mathrm{CA}+2.3\mathrm{FA}+4.94W+8.9\right)\;+(0.000124C(\mathrm{CA}+\mathrm{FA}))/A$$

$$\sigma_{2}=3.19\log({\mathrm{AS}}^{3}/C^{3}{\left(C-7.2\right)}^{3}{\left(\mathrm{AS}+2.0C\right)}^{3}))\;$$

$$\sigma_{3}=133\mathrm{SP}-133\mathrm{CA}/(2\mathrm{SP}-2W)(W/\log(W)-7.2)\;$$

$$\sigma_{4}=-0.00464\mathrm{SP}\left(W+\log({\mathrm{AS}}^{3}\right)+\frac{\mathrm{FA}}{A}-7.2{)}^{\frac{1}{2}}\left(\mathrm{CA}+\mathrm{FA}-3\mathrm{SP}+2W-\frac{\mathrm{FA}\;\log\left(\mathrm{CA}\right)}{\mathrm{SP}-W}\right)$$

**Figure A1:**
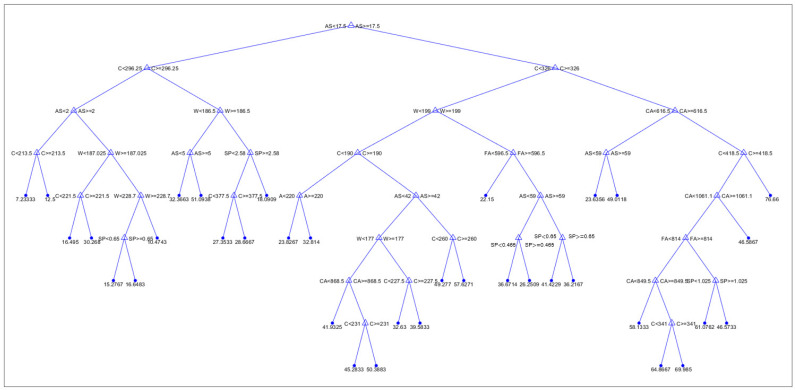
Optimal individual model based on regression trees.

**Figure A2:**
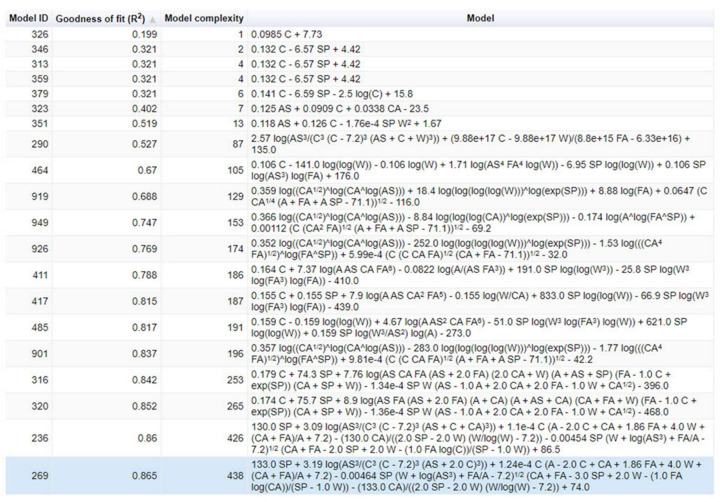
MGGP models that form the Pareto front.

For the methods based on decision trees, the application of both individual tree models and models based on ensembles of individual models was analyzed. In the case of individual models (Figure A1), trees of different depths were considered, which were defined by the limitation in terms of the minimum number of data per terminal leaf, and all individual models were evaluated in terms of the defined accuracy criteria. An optimal model (Figure A1) with a limit of at least six data per terminal leaf was obtained (Table 5).

**Figure 13:**
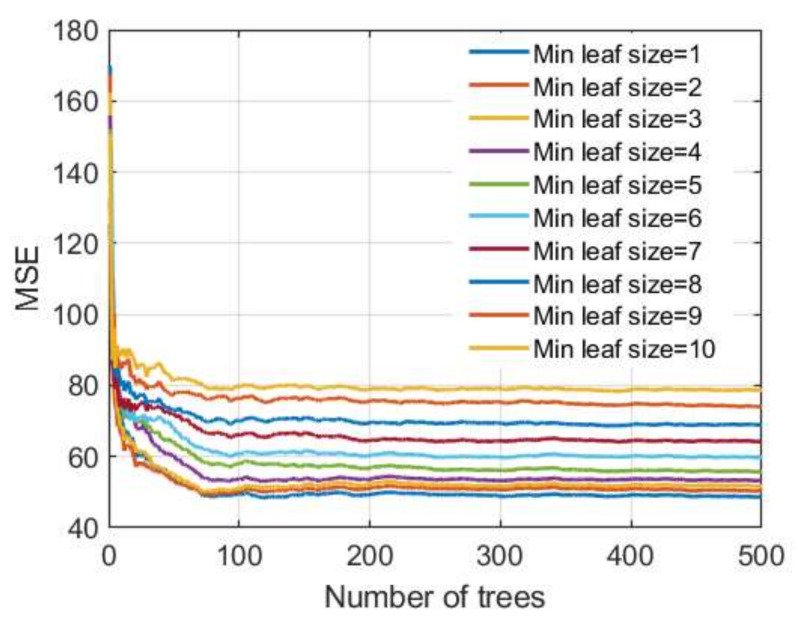
MSE vs. number of trees in TreeBagger model for different minimum leaf sizes.

**Figure 14:**
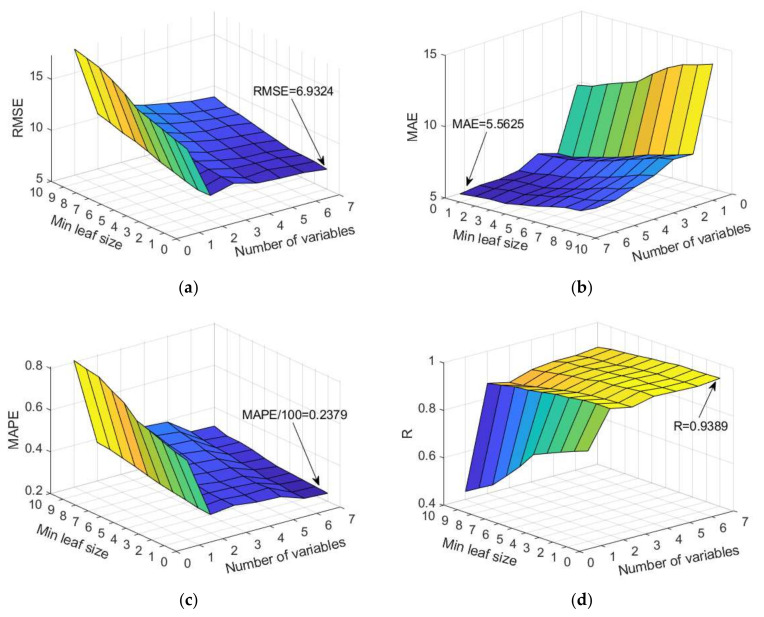
Comparison of different accuracy criteria for RF model as a function of the number of randomly selected splitting variables and minimum leaf size: (**a**) RMSE, (**b**) MAE, (**c**) MAPE, and (**d**) R.

When applying the TreeBagger (Figure 13) and RF (Figure 14) methods based on decision trees, the following adaptive parameters were considered:

1. Number of generated trees B;
2. The minimum value of a leaf size (min leaf size) that represents the minimum amount of data assigned to a leaf within the tree;
3. Number of randomly selected variables from the whole set of variables on which tree splitting will be performed (RF method only).

During the formation of the ensemble, 500 different datasets were generated from the original dataset, and then a model was created on each of these generated sets. The TreeBagger method uses all input variables when creating a model.

In the implementation of RF, it is recommended that the number of randomly selected variables be approximately equal to one-third of the total number of variables, which in most problems should lead to a satisfactory model. Such assumptions have been adopted in the program implementation in the MATLAB 2020a program (default settings) [21,25]. In this case, it would mean the adoption of models which generate regression trees using two or three variables out of the seven variables, which is not fully accepted in this study. A significant number of models were analyzed using a randomly selected subset of 2, 3, 4, 5, and 6 variables, and the analysis included the evaluation of the model using RMSE, MAE, R, and MAPE criteria.

The analysis of a specific dataset showed that the usually recommended values of a random selection of a subset of two or three variables according to which tree splitting would be performed are not optimal. Additionally, the default setting of a minimum number of five data per terminal leaf is not optimal in terms of defined criteria. According to the three criteria RMSE, MAE, and MAPE, the selection of the whole set of variables when creating trees gives the model significantly higher accuracy, while with regard to the criterion R, this difference is less pronounced. Furthermore, the analysis showed that, in this case, the creation of deep trees (min leaf size = 1) provides models with greater accuracy in terms of all criteria. The case of selecting the whole set of seven input variables in Figure 14 corresponds to the TreeBagger model, which for the given case gives the highest accuracy of prediction.

Regarding the number of trees that make up the ensemble in the TreeBagger model, it can be seen from Figure 13 that a significant number of basic models is needed to achieve the correct accuracy and that the learning curve is saturated at approximately 250 basic models.

**Figure 15:**
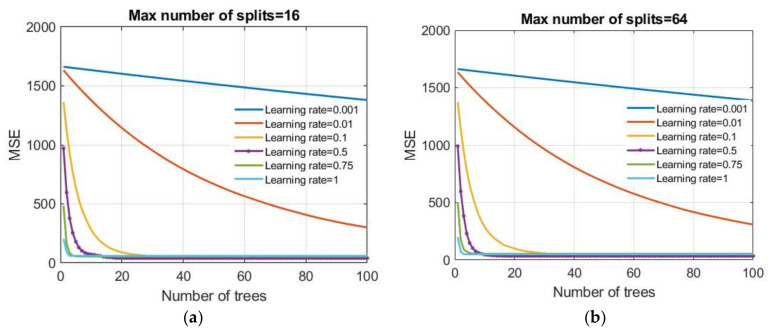
Dependence of the MSE value on the reduction parameter λ and the number of trees (base models) in the boosted trees model: (**a**) the maximum number of splits is limited to 16 and (**b**) the maximum number of splits is limited to 64.

The boosting trees method combines binary regression trees using a gradient boosting technique. Creating a model goes sequentially. The following model parameters (Figure 15) were analyzed:

- Number of generated trees B;
- Reduction parameter λ (learning rate);
- Number of splits of the tree d.

A number of values of reduction parameters were investigated in this paper as follows: 0.001; 0.01; 0.1; 0.5; 0.75, and 1.0. Additionally, tree models were generated that had restrictions on the maximum number of splits of $2^{0}=1,\;2^{1},2^{2},\;2^{3},2^{4},\;2^{5},2^{6},\;2^{7},\;2^{8}=256.$

The MSE criterion was used as the criterion of optimality. After model training, all obtained models were analyzed using RMSE, MAE, MAPE, and R criteria on a test dataset. In terms of defined criteria, two models stood out: model 1 (boosted trees 1 model) whose parameters are the max number of splits d = 16 and learning rate = 0.5 which is optimal in terms of RMSE and R criteria; model 2 (boosted trees 2 model) with parameters being the max number of splits d = 64 and learning rate = 0.5, which are optimal in terms of MAE and MAPE criteria. For both models, the minimum number of data per terminal leaf is five.

**Figure 16:**
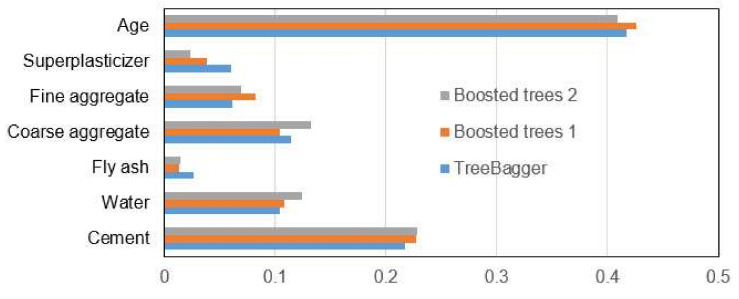
Significance of individual variables in TreeBagger and boosted trees models.

With all ensemble models, it is possible to see the importance of individual input variables on the accuracy of the model. This analysis was performed on the Treebager model and the boosted trees models. The obtained values are displayed in Figure 16.

When applying the SVM method, the application of three different kernels was analyzed: linear, RBF, and sigmoid kernel.

When preparing data for all analyzed models, all input values were transformed into the range [0, 1], i.e., linear data transformation was performed. The values of the output target vector were also scaled into same range. When estimating the model, the values of all variables were rescaled and the performance of the model was determined using accuracy criteria. The RMSE value as the optimality criterion was used for the selection of parameters in all models. The process of finding the optimal parameters of the model was implemented using LIBSVM [56] through two phases, the rough search phase and the detailed search phase, using the grid search method.

In the case of a linear kernel function, there are no unknown parameters of the function itself, and the use of the function was reduced to finding the values of the scalar products of the input vectors. However, for the development of the model, it was necessary to determine the value of C of the penalty parameter as well as the values of the insensitivity zone of model ε .

For the RBF kernel, within the function itself, it was necessary to determine parameter γ (the width of the Gaussian curve bell), the value of C of the penalty parameter, and the values of the insensitivity zone ε .

In the sigmoid kernel function, in the general case, two unknown parameters γ and r appear; in addition to the development of the model, it was necessary to determine the value of C of the penalty parameter and the values of the insensitivity zone of the model ε . In accordance with the recommendation in the paper [52], it is assumed that the value of the parameter r is equal to zero.

A similar procedure with the appropriate range of values was applied to all analyzed kernels in the rough parameter search phase:

- When applying the linear kernel in this study, the values for parameter C in exponential order in the range $C=\left[2^{-10},2^{-9},\ldots ,2^{0},\ldots ,2^{9},2^{10}\right]$ were examined as well as the values for the parameter ε in the range $\varepsilon =\left[2^{-10},2^{-9},\ldots ,2^{0},2^{1}\right]$ ;
- For the RBF kernel, the values for parameter C in the exponential order in the range $C=\left[2^{-10},2^{-9},\ldots ,2^{0},\ldots ,2^{9},2^{10}\right]$ , the values of *γ* in the range $\gamma =\left[2^{-10},2^{-9},\ldots ,2^{0},\ldots ,2^{9},2^{10}\right]$ , as well as the values for the parameter ε in the range $\varepsilon =\left[2^{-10},2^{-9},\ldots ,2^{0},2^{1}\right]$ were examined;
- For the sigmoid kernel, the values for the parameter C in the exponential order in the range $C=\left[2^{-10},2^{-9},\ldots ,2^{0},\ldots ,2^{9},2^{10}\right]$ , γ in the range $\gamma =\left[2^{-10},2^{-9},\ldots ,2^{0},\ldots ,2^{9},2^{10}\right]$ , as well as the values for the parameter ε in the range $\varepsilon =\left[2^{-10},2^{-9},\ldots ,2^{0},2^{1}\right]$ were examined. A value of zero was adopted for the value of the parameter r ( r=0 ) in accordance with the recommendation [56];
- All possible combinations of parameters within the above ranges were analyzed.

In the phase of detailed search, a narrow search was performed in the area of the obtained optimal solution of rough search. The procedure was limited to 100 iterations. During each iteration, the value of the search steps decreased. The lower limit of the search area was obtained by decreasing the optimal value of the parameter from the previous iteration by the value of the steps from the previous iteration, and the upper limit by increasing the optimal value from the previous iteration by the value of the steps from the previous iteration. The procedure is repeated until the specified number of iterations is reached (Table 6).

In GPR, kernel functions determine the covariance between two latent variables $f\left(x_{i}\right)$ and $f\left(x_{j}\right)$ where both vectors $x_{i}$ and $x_{j}$ are dimensions *d* × 1. Kernel functions show how much the output or response of the system at some point $x_{i}$ is affected by the response of the system at some point $x_{j}$ , i≠j, j=1,2,…,n .

The influence of the covariance function $k\left(x_{i},x_{j}\right)$ in this paper was examined using different functions that are parameterized, using standard signal deviation $\sigma_{f}$ (output) and one length scale parameter $\sigma_{l}$ or more length scale parameters $\sigma_{l},\;l=1,2,\ldots ,m$ if different parameters of the distance scale for different coordinate axes for ARD functions are analyzed.

The data for model development are first standardized in the paper. By the standardization procedure, which was carried out by columns, the data were transformed to have a mean value of zero and a variance equal to one. The application of the model with constant base function was analyzed. In the process of finding the optimal GPR model, two groups of covariance functions were analyzed, namely:

1. Covariance functions that have one length scale parameter for all input variables (exponential, quadratic-exponential, Matérn 3/2, Matérn 5/2, rational square);
2. Covariance functions that apply different length scale parameters to input variables (ARD covariance functions).

The parameters of the analyzed covariance functions were determined using gradient procedures on the expression for log marginal probability.

Analysis of the application of a total of 10 different covariance functions (Table 7 and Table 8) showed that more accurate models are obtained by applying ARD functions. The model with ARD exponential function proved to be the optimal model, which is better than other models in terms of the three defined criteria RMSE, MAE, and R. In terms of criteria, MAPE is the second-ranked model, but the difference is 0.006647 compared to the first-ranked one and can practically be ignored.

**Figure 17:**
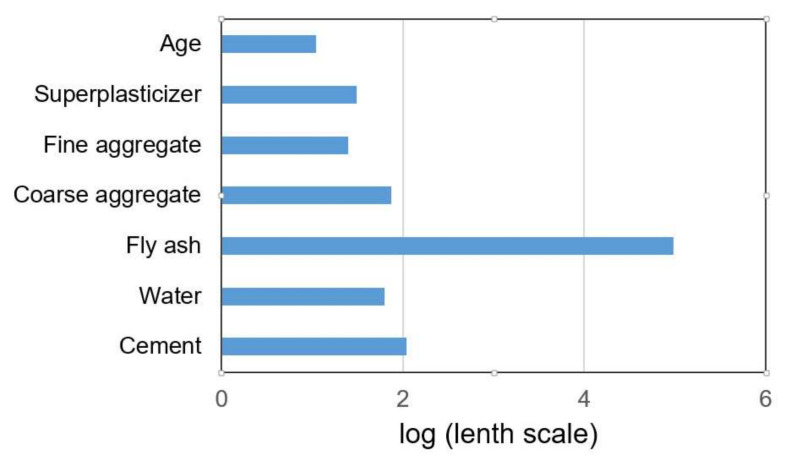
Variable importance using optimal ARD exponential covariance function.

The values of the length scale parameters for individual input variables can be used to assess the significance of these variables in terms of their impact on model accuracy. The significance of individual variables is inversely proportional to the size of these parameters. For the optimal model with the ARD SE covariance function, the logarithms (with base 10) of the length scale parameters are shown in Figure 17.

In the application of ANN models, different models of MLP neural network with one hidden layer were investigated, wherein neurons with a tan sigmoid activation function in the hidden layer were used, and neurons with a linear activation function were used in the output layer.

A value of 15 neurons was adopted for the value of the upper limit of the number of neurons in the hidden layer,

$$N_{H}\leq min\left(2\times 7+1=15,\;\frac{327}{7+1}=40.9\right)=15.$$

All model variables were transformed into the interval [−1, 1], where the corresponding minimum value was mapped to -1 and the corresponding maximum value to 1, and linear scaling was used for the values in between. The setting of the model parameters is shown in Table 9.

**Figure 18:**
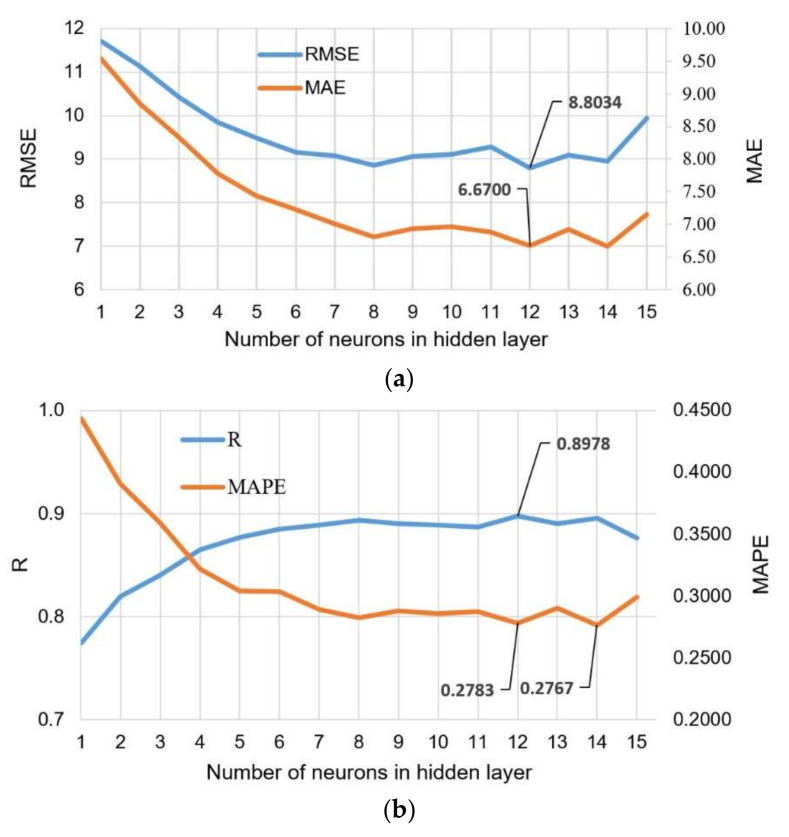
Comparison of the accuracy criteria for MLP-ANNs with different numbers of neurons in the hidden layer: (**a**) RMSE and MAE; and (**b**) R and MAPE.

The architecture of the input and output layer is defined by the problem being modeled, i.e., in this case, the number of neurons in the input layer is seven, and the number of output layers is one. The optimal number of neurons in the hidden layer according to the 3 criteria RMSE, MAE, and R is 12 (Figure 18). The model that has 14 neurons in the hidden layer is optimal according to the MAPE criterion, but this difference compared to the previously mentioned model is only 0.0016.

**Figure 19:**
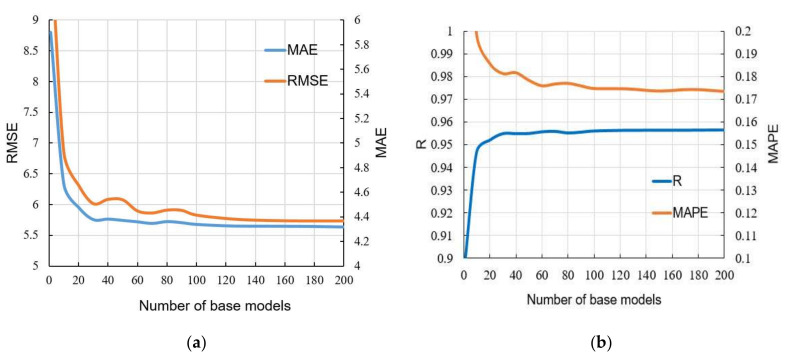
Comparison of accuracy criteria for the ensemble with a different number of individual ANN models within the ensemble: (**a**) RMSE and MAE; and (**b**) R and MAPE.

**Figure 20:**
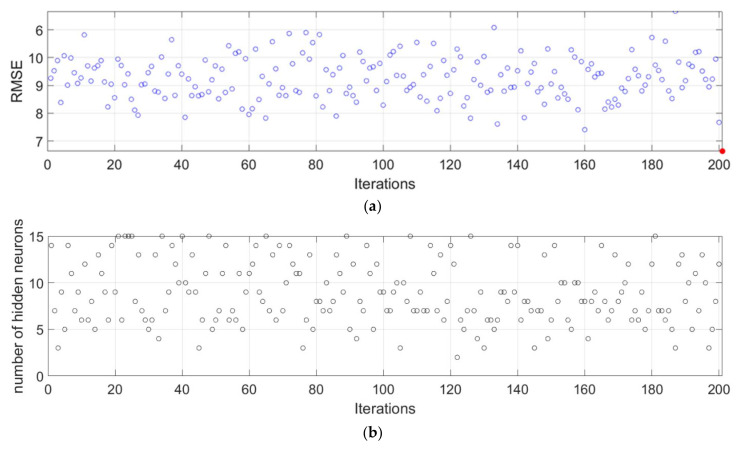
(**a**) RMSE value for each of the iterations; and (**b**) the optimal number of neurons in the hidden layer in each of iteration.

**Figure 21:**
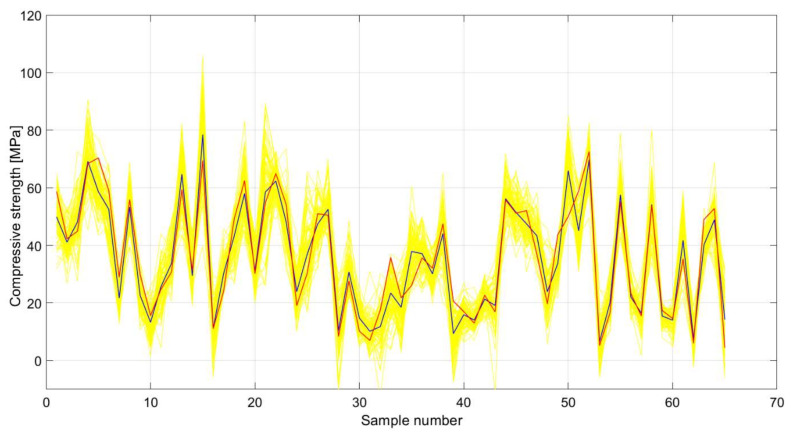
Comparative analysis of anensemble of ANNs (blue color) and individual ANN models (yellow color) in relation to target test values (red color) for SCC with Class F fly ash compressive strength.

Using bootstrap sampling with replacement, a large number of different datasets from the original set were formed, and then individual models of ANNs were formed over these datasets. As such, an ensemble of ANNs was formed, the prediction of which is the average value of individual models. With the formation of the ensemble, all criteria for the accuracy of the model (Figure 19, Figure 20 and Figure 21) were significantly improved. The comparison of all analyzed machine learning models is given in Table 10.

The selection of the optimal model in Table 10 was made on the basis of four criteria. The RMSE and MAE criteria are absolute indicators of the accuracy of the model and are expressed in the same units as the output variable, which in this case is MPa. The RMSE criterion is a criterion that is more representative of the accuracy of the model in relation to the extreme values within the dataset, while the MAE is an indicator of the absolute accuracy of the model. The R and MAP criteria are relative criteria that are dimensionless. In this particular case, the ensemble model can be considered optimal because it achieves the best values of three indicators compared to the four defined. The optimal model consists of individual models created in iterations. In each iteration, a sample of the same size as the original is formed using bootstrap aggregation, different architectures that can have up to 15 neurons are examined, and a model with the optimal architecture in the current iteration joins the ensemble. This process is repeated iteratively until the learning curve is saturated. As such, the accuracy of an individual model is significantly increased.

## 5. Conclusions

This paper describes a number of state-of-the-art machine learning methods that can be used to predict CS in SCC with the addition of Class F fly ash. The paper discusses the application of MGGP, RT, and ensembles based on RTs, TreeBagger, random forest, and boosted tree models, SVM models with linear, RBF, and sigmoid kernels, GPR models, models of individual ANN, and ensembles composed of individual ANN models.

This paper presents a detailed procedure for determining the optimal parameters for all analyzed models and determines the accuracy of each model in terms of the defined criteria of RMSE, MAE, R, and MAPE. The analyzed models indicated the importance of individual input variables on the accuracy of the model and the positive effect of fly ash on the CS of concrete.

Based on a respectable database of performed tests from the literature, both individual models and ensemble models were analyzed and the accuracy in terms of defined criteria was assessed. The advantage of using an ensemble of neural networks and GPR models that were not used in the discussions in the cited literature was pointed out. In addition, less accurate but more transparent individual MGGP and RT models were analyzed and compared to widely used ANN models. Both models provide the relationship of individual constituents with the compressive strength of SCC concrete. The MGGP model provides this relationship with the aid of an equation while the RT model provides this relationship in the form of a simple tree structure, which in some cases, is important for operational implementation. Findings in the paper allow several industries to model their own mix in less time without any money invested in it. In terms of the three defined criteria, RMSE, MAPE, and R, the model of an ensemble of ANNs stood out as an optimal model with values of 5.6351 MPa, 4.3665 MPa, and 0.9563, respectively. As this is a model that predicts the SC for different ages of concrete from 1 to 365 days, the achieved accuracy of the model can be considered satisfactory. The application of bootstrap aggregation led to a significant increase in accuracy compared to the individual ANN model. The expansion of the database could further improve the accuracy of the obtained model.

GPR models with the ARD exponential function showed quite similar and slightly worse accuracy, with a value of 4.4334 MPa for MAE criteria. This model, thanks to the ARD property, has the ability to determine the significance of individual variables on the accuracy of the prediction model.

From the individual analyzed models, the MGGP model could be singled out as useful because the model provides an explicit expression that can be used in the prediction of concrete of different ages. The accuracy in terms of defined absolute criteria RMSE and MAE is 1.46 MPa and 1.33 MPa less than the optimal ensemble of neural networks model, respectively. The defined RT model, although much simpler, gave almost the same accuracy as the optimal individual NN model.

## Figures and Tables

**Table 1:** Review of the application of ML algorithms in the development of compressive strength (CS) prediction models in self-compacting concrete (SCC).

Type of Concrete	Algorithm	Data Points	Year	Authors	Reference
SCC	ANN	80	2011	Siddique et al.	[7]
SCC	ANN	169	2016	Asteris et al.	[8]
SCC	ANN	114	2016	Douma et al.	[9]
SCC	ANN	205	2017	Asteris et al.	[10]
SCC	Support vector machines (SVM)	115	2019	Saha et al.	[11]
SCC	SVM	340	2019	Azimi-Pour et al.	[12]
Lightweight self-compacting concrete (LWSCC)	Beetle antennae search (BAS)-algorithm-based random forest (RF)	131	2019	Zhang et al.	[13]
SCC	Regression trees (RT), gene-expression programming (GEP), boosting regressor (BR)	97	2021	Song et al.	[14]
Self-compacting concrete with waste rubber (SCRC)	Gaussian process regression (GPR)	144	2021	Hadzima-Nyarko et al.	[15]
Self-compacting concrete with waste rubber (SCRC)	NN, Bagged trees, RF, boosted trees, SVM, GPR	166	2021	Kovacevic et al.	[16]
SCC	NN, NN + firefly optimization algorithm (FOA)	327	2021	Pazouki et al.	[17]
SCC	NN, SVM, GEP	300	2021	Farooq et al.	[18]
SCC with recycled aggregates	RF, KNN, ERT, XGB, GB, LGBM, CB, GAM	515	2022	de-Prado-Gil et al.	[19]

**Table 2:** Statistical properties of variables used for modeling.

Statistical Analysis of Input and Output Parameters for **All Data**						
**Constituent**	**Max.**	**Min.**	**Mean**	**Mode**	**St.Dev.**	**Count**
Cement (C) (kg/m^3^)	503	61.00	293.08	250.00	89.78	327
Water (W) (kg/m^3^)	390.39	132.00	197.00	180.00	37.62	327
Fly ash (A) (kg/m^3^)	373.00	20.00	170.23	160.00	69.68	327
Coarse aggregate (CA) (kg/m^3^)	1190.00	590.00	828.34	837.00	137.30	327
Fine aggregate (FA) (kg/m^3^)	1109.00	434.00	807.47	910.00	135.80	327
Superplasticizer (SP) (%)	4.60	0	0.980	0.50	1.11	327
Age of samples (AS) (days)	365	1	44.31	28.00	63.76	327
Compressive strength (MPa)	90.60	4.44	36.45	12.00	19.07	327
Statistical analysis of input and output parameters for **Training set**						
	Max.	Min.	Mean	Mode	St.Dev.	Count
Cement (C) (kg/m^3^)	503.00	61.00	292.57	250.00	86.29	262
Water (W) (kg/m^3^)	390.39	133.20	197.83	180.00	39.09	262
Fly ash (A)(kg/m^3^)	373.00	20.00	169.40	160.00	67.80	262
Coarse aggregate (CA) (kg/m^3^)	1190.00	590.00	825.08	837.00	129.38	262
Fine aggregate (FA) (kg/m^3^)	1109.00	434.00	811.44	910.00	128.91	262
Superplasticizer (SP) (%)	4.60	0	0.98	0.50	1.10	262
Age of samples (AS) (days)	365	1	43.86	28.00	62.63	262
Compressive strength (MPa)	90.60	4.90	36.55	12.00	19.05	262
Statistical analysis of input and output parameters for **Test set**						
Cement (C) (kg/m^3^)	503.00	61.00	295.11	295.11	103.36	65
Water (W) (kg/m^3^)	279.50	132.00	193.68	193.68	31.07	65
Fly ash (A)(kg/m^3^)	336.00	20.00	173.56	173.56	77.27	65
Coarse aggregate (CA) (kg/m^3^)	1190.00	590.00	841.48	841.48	165.95	65
Fine aggregate (FA) (kg/m^3^)	1109.00	434.00	791.43	791.43	160.74	65
Superplasticizer (SP) (%)	4.60	0	0.98	0.98	1.15	65
Age of samples (AS) (days)	365	1	46.09	46.09	68.61	65
Compressive strength (MPa)	72.61	4.44	36.02	4.44	19.32	65

**Table 3:** Setting parameters for MGGP models.

Parameter	Setting
Function set	times, minus, plus, rdivide, square, exp, log, mult3, sqrt, cube, power
Population size	100
Number of generations	1000
Max number of genes	6
Max tree depth	7
Tournament size	10
Elitism	0.05% of population
Crossover probability	0.85
Mutation probability	0.1
Probability of Pareto tournament	0.5

**Table 4:** Comparison of performance measures for MGGP models.

Model ID	RMSE	MAE	MAPE	R
901	8.3826	6.3119	0.2437	0.9065
316	8.6407	6.5785	0.2578	0.9038
320	7.9860	6.2587	0.2444	0.9175
236	7.2654	5.7730	0.2411	0.9294
269	**7.0962**	**5.6960**	**0.2409**	**0.9295**

**Table 5:** Influence of minimum leaf size on regression tree (RT) model accuracy.

Min Leaf Size	RMSE	MAE	MAPE	R
1	9.5144	7.2558	0.2687	0.8873
2	9.5368	7.3382	0.2821	0.8780
3	9.0359	6.9911	0.2627	0.8873
4	9.4848	7.3640	0.2753	0.8775
5	9.6157	7.3791	0.2769	0.8747
6	**8.8407**	**6.6424**	**0.2253**	**0.8942**
7	9.7815	6.9879	0.2458	0.8685
8	10.1599	7.3942	0.2640	0.8569
9	10.9231	8.4503	0.3106	0.8283
10	10.7970	8.4804	0.3232	0.8312

**Table 6:** Values of optimal kernel function parameters in the SVM method.

SVM linear	C=0.1428	ε=0.0436	/
SVM RBF	C=17.6919	ε=0.0412	γ=2.1220
SVM sigmoid	C=252.3998	ε=0.0547	γ=9.1574

**Table 7:** Values of optimal parameters in GPR models with different covariance functions.

GP Model Covariance Function	Covariance Function Parameters		
Exponential	$k\left(\left(x_{i},x_{j}|\Theta \right)\right)=\sigma_{f}^{2}exp\left[-\frac{1}{2}\frac{r}{\;\sigma_{l}{}^{2}}\right]$		
	$\sigma_{l}=$ 31.24		$\sigma_{f}=$ 54.56
Squared exponential	$k\left(\left(x_{i},x_{j}|\Theta \right)\right)=\sigma_{f}^{2}exp\left[-\frac{1}{2}\frac{{\left(x_{i}-x_{j}\right)}^{T}\left(x_{i}-x_{j}\right)}{\;\sigma_{l}{}^{2}}\right]$		
	$\sigma_{l}=$ 1.71		$\sigma_{f}=$ 29.25
Matérn 3/2	$k\left(\left(x_{i},x_{j}|\Theta \right)\right)=\sigma_{f}^{2}\left(1+\frac{\sqrt{3}r}{\sigma_{l}}\right)exp\left[-\frac{\sqrt{3}r}{\sigma_{l}}\right]$		
	$\sigma_{l}=$ 4.17		$\sigma_{f}=$ 41.74
Matérn 5/2	$k\left(\left(x_{i},x_{j}|\Theta \right)\right)=\sigma_{f}^{2}\left(1+\frac{\sqrt{5}r}{\sigma_{l}}+\frac{5r^{2}}{3\sigma_{l}{}^{2}}\right)exp\left[-\frac{\sqrt{5}r}{\sigma_{l}}\right]$		
	$\sigma_{l}=$ 2.68		$\sigma_{f}=$ 34.12
Rational quadratic	$k\left(\left(x_{i},x_{j}|\Theta \right)\right)=\sigma_{f}^{2}{\left(1+\frac{r^{2}}{2a\sigma_{l}{}^{2}}\right)}^{-\alpha };r=0$		
	$\sigma_{l}=$ 3.05	a= 0.22	$\sigma_{f}=$ 47.62

where $r=\sqrt{{\left(x_{i}-x_{j}\right)}^{T}(x_{i}-x_{j})}$ .

**Table 8:** Values of optimal parameters in GPR ARD models with different covariance functions.

Covariance Function Parameters						
$\sigma_{1}$	$\sigma_{2}$	$\sigma_{3}$	$\sigma_{4}$	$\sigma_{5}$	$\sigma_{6}$	$\sigma_{7}$
ARD exponential: $k\left(\left(x_{i},x_{j}|\Theta \right)\right)=\sigma_{f}^{2}\exp\left(-r\right)$ $;\;\sigma_{F}$ $=64.78;\;r=\sqrt{\overset{d}{\underset{m=1}{\sum }}\frac{{\left(x_{im}-x_{jm}\right)}^{2}}{\;\sigma_{m}{}^{2}}}$						
109.27	61.97	97,176.47	74.09	24.72	31.09	10.95
ARD squared exponential: $k\left(\left(x_{i},x_{j}|\Theta \right)\right)=\sigma_{f}^{2}exp\left[-\frac{1}{2}\overset{d}{\underset{m=1}{\sum }}\frac{{\left(x_{im}-x_{jm}\right)}^{2}}{\;\sigma_{m}{}^{2}}\right];$ $\sigma_{f}$ = 32.48						
4.12	3.35	7.92	6.31	3.29	0.48	1.12
ARD Matérn 3/2: $k\left(\left(x_{i},x_{j}|\Theta \right)\right)=\sigma_{f}^{2}\left(1+\sqrt{3}r\right)exp\left[-\sqrt{3}r\right];$ $\sigma_{f}$ = 25.12						
4.61	2.33	5649.90	3.39	0.97	1.37	0.48
ARD Matérn 5/2: $k\left(\left(x_{i},x_{j}|\Theta \right)\right)=\sigma_{f}^{2}\left(1+\sqrt{5}r+\frac{5r^{2}}{3}\right)exp\left[-\sqrt{5}r\right];$ $\sigma_{f}$ = 30.34						
4.23	4.86	8.88	6.91	3.99	0.70	0.53
ARD rational quadratic: $k\left(\left(x_{i},x_{j}|\Theta \right)\right)=\sigma_{f}^{2}{\left(1+\frac{1}{2\alpha }\overset{d}{\underset{m=1}{\sum }}\frac{{\left(x_{im}-x_{jm}\right)}^{2}}{\;\sigma_{m}{}^{2}}\right)}^{-\alpha };\;\alpha$ $=30.34;\;\;\sigma_{f}$ = 51.96						
4.45	1.97	18,417.04	4.87	1.08	5.89	0.57

where $r=\sqrt{\sum_{m=1}^{d}\frac{{\left(x_{im}-x_{jm}\right)}^{2}}{\;\sigma_{m}{}^{2}}}$ .

**Table 9:** Parameter settings applied during ANN model calibration in MATLAB.

Parameter	Parameter Value	
	Lower Limit	Upper Limit
Number of epochs	/	1000
MSE value (performance)	/	0
Gradient	/	1.00 × 10^−7^
$\mathrm{The}\;\mathrm{value}\;\mathrm{of}\;\mathrm{the}\;\mathrm{parameter}\;\lambda_{k}$ (Mu)	0.005	1.00 × 10^10^

**Table 10:** Comparative analysis of results of different machine learning models.

Model	RMSE	MAE	MAPE/100	R
MGGP	7.0962	5.6960	0.2409	0.9295
Decision tree	8.8407	6.6424	0.2253	0.8942
TreeBagger	7.0236	5.5892	0.2387	0.9378
Random forest	6.9324	5.5627	0.2379	0.9389
Boosted tree 1	5.9597	4.8307	0.1793	0.9518
Boosted tree 2	6.3814	4.5410	0.1580	0.9237
SVM linear	12.7268	10.6332	0.5323	0.7495
SVM RBF	5.9533	4.5551	0.1976	0.9521
SVM sigmoid	12.6875	10.4926	0.5242	0.7511
GP exponential	6.7391	5.3043	0.2313	0.9376
GP Sq.exponential	6.5298	5.0244	0.2117	0.9429
GP Matérn 3/2	6.3409	4.7695	0.1909	0.9454
GP Matérn 5/2	6.3686	4.7852	0.1943	0.9452
GP Rat. quadratic	6.4138	4.8693	0.1970	0.9439
GP ARD exponential	5.9891	4.4334	0.1625	0.9517
GP ARD Sq. exponential	6.2278	4.6602	0.1739	0.9506
GP ARD Matérn 3/2	6.2476	4.6911	**0.1559**	0.9481
GP ARD Matérn 5/2	6.4760	4.5837	0.1632	0.9463
GP ARD Rat. quadratic	6.2025	4.6228	0.1560	0.9494
ANN	8.8034	6.6700	0.2783	0.8978
Ensemble ANN	**5.6351**	**4.3665**	0.1734	**0.9563**

## Data Availability

Not applicable.

## Supplementary Materials

The following are available online at https://www.mdpi.com/1996-1944/15/12/4191/s1, The complete database of tests of SCC samples (Supplementary Materials S1).
