# Supplementary material for: Application of Artificial Intelligence Methods for Predicting the Compressive Strength of Self-Compacting Concrete with Class F Fly Ash
Source: Materials (Basel). 2022 Jun 13;15(12):4191. doi: 10.3390/ma15124191 (PMC9229836; doi:10.3390/ma15124191)
Supplement: Supplementary file 1 [file materials-15-04191-s001.zip › DatabaseFlyAsh/Database.pdf]

materials

MDPI

Supplementary materials

Application of Artificial Intelligence Methods for Predicting the Compressive Strength of Self-Compacting Concrete with Class F Fly Ash

Miljan Kovačević [1*], Silva Lozančić [2*], Emmanuel Karlo Nyarko [3] and Marijana Hadzima-Nyarko [2]

1 Faculty of Technical Sciences, University of Pristina, Knjaza Milosa 7, 38220 Kosovska Mitrovica, Serbia
2 Faculty of Civil Engineering and Architecture Osijek , Josip Juraj Strossmayer University of Osijek, Vladimira Preloga 3, 31000 Osijek, Croatia; mhadzima@gfos.hr
3 Faculty of Electrical Engineering, Computer Science and Information Technology, Josip Juraj Strossmayer University of Osijek, Kneza Trpimira 2B, 31000 Osijek, Croatia; karlo.nyarko@ferit.hr
* Correspondence: miljan.kovacevic@pr.ac.rs (M.K.); lozancic@gfos.hr (S.L.); Tel.: +381-606173801 (M.K.); +385-31-544-665 (S.L.)

Training dataset

Sample	C (kg/m^3)	W (kg/m^3)	Class F FA (kg/m3)	CA (kg/m3)	FA (kg/m3)	SP (%)	Age (days)	f'c (MPa)
1	350.00	251.16	133.00	883.00	815.00	0.16	28.00	55.30
2	225.00	175.00	275.00	652.00	908.00	1.50	7.00	40.85
3	300.00	190.00	200.00	735.00	910.00	1.35	7.00	27.94
4	232.00	135.45	155.00	847.00	846.00	0.38	28.00	48.30
5	350.00	190.00	150.00	735.00	910.00	1.35	7.00	33.57
6	375.00	195.00	125.00	735.00	910.00	1.35	7.00	30.37
7	160.00	156.00	240.00	900.00	886.00	0.35	28.00	44.00
8	260.00	227.90	170.00	837.00	717.00	0.70	7.00	16.70
9	250.00	225.50	160.00	837.00	739.00	0.00	90.00	42.00
10	260.00	227.90	170.00	837.00	717.00	0.70	90.00	38.20
11	260.00	215.00	260.00	775.00	757.00	0.77	28.00	30.90
12	355.00	242.00	195.00	590.00	910.00	1.80	90.00	40.84
13	220.00	176.00	330.00	881.00	686.00	1.21	90.00	64.80
14	350.00	214.00	170.00	792.00	774.00	0.81	365.00	74.10
15	220.00	156.00	180.00	900.00	916.00	0.35	1.00	13.00
16	280.00	258.50	190.00	837.00	599.00	0.70	90.00	36.50
17	405.00	189.00	45.00	612.00	1109.00	4.60	28.00	15.99
18	198.00	154.80	232.00	900.00	872.00	0.50	1.00	13.00
19	250.00	225.50	160.00	837.00	742.00	0.50	28.00	26.96
20	375.00	195.00	125.00	735.00	910.00	1.35	130.00	53.56
21	183.00	188.65	160.00	837.00	891.00	0.50	28.00	22.10
22	170.00	159.10	200.00	900.00	928.00	0.50	28.00	33.00
23	400.00	180.00	20.00	876.00	845.00	0.70	7.00	44.00
24	360.00	189.00	90.00	612.00	1109.00	4.60	7.00	15.00
25	250.00	225.50	160.00	837.00	746.00	1.00	90.00	40.30
26	300.00	200.00	200.00	860.00	845.00	1.80	90.00	35.00

Sample	C (kg/m^3)	W (kg/m^3)	Class F FA (kg/m3)	CA (kg/m3)	FA (kg/m3)	SP (%)	Age (days)	f'c (MPa)
27	220.00	180.00	180.00	900.00	850.00	0.35	1.00	8.00
28	450.00	188.00	70.00	844.00	825.00	2.34	90.00	78.60
29	380.00	200.00	192.00	621.00	931.00	0.43	7.00	45.70
30	350.00	214.00	170.00	792.00	774.00	0.81	28.00	50.30
31	325.00	190.00	175.00	735.00	910.00	1.35	3.00	22.36
32	325.00	190.00	175.00	735.00	910.00	1.35	130.00	58.11
33	250.00	155.80	160.00	837.00	919.00	0.50	7.00	23.90
34	225.00	189.00	225.00	612.00	1109.00	4.60	7.00	12.09
35	349.00	291.27	162.00	852.00	779.00	0.14	28.00	59.90
36	250.00	155.80	160.00	837.00	919.00	0.50	90.00	48.30
37	230.00	192.40	140.00	837.00	864.00	0.80	90.00	43.50
38	220.00	156.00	180.00	900.00	916.00	0.35	1.00	12.00
39	225.00	180.00	225.00	818.00	818.00	0.35	7.00	26.20
40	440.00	176.00	110.00	917.00	714.00	1.35	28.00	69.80
41	240.00	202.80	150.00	837.00	831.00	0.60	90.00	44.20
42	280.00	258.50	190.00	837.00	599.00	0.70	7.00	14.70
43	337.50	189.00	112.50	612.00	1109.00	4.60	7.00	19.66
44	325.00	250.25	60.00	850.00	899.00	0.43	28.00	31.70
45	250.00	225.50	160.00	837.00	742.00	0.50	90.00	41.50
46	275.00	351.75	250.00	840.00	775.00	0.09	28.00	54.50
47	250.00	390.39	257.00	853.00	787.00	0.11	28.00	51.50
48	290.00	229.50	220.00	837.00	625.00	0.20	90.00	44.90
49	237.00	133.20	133.00	900.00	1034.00	0.20	28.00	49.00
50	198.00	153.00	232.00	900.00	874.00	0.20	1.00	9.00
51	207.00	186.30	207.00	843.00	845.00	0.40	1.00	6.10
52	415.00	231.00	135.00	590.00	910.00	1.80	365.00	46.57
53	317.00	262.35	160.00	837.00	594.00	0.50	90.00	29.10
54	237.00	159.10	133.00	900.00	960.00	0.50	28.00	46.00
55	375.00	175.00	125.00	679.00	938.00	1.50	3.00	57.26
56	180.00	166.50	270.00	850.00	850.00	0.21	1.00	5.00
57	380.00	200.00	192.00	621.00	931.00	0.43	1.00	23.20
58	333.00	173.00	162.00	924.00	743.00	0.75	90.00	63.00
59	250.00	281.05	261.00	837.00	478.00	0.50	90.00	29.50
60	400.00	180.00	20.00	876.00	845.00	0.70	56.00	63.11
61	247.00	185.40	165.00	846.00	845.00	0.12	28.00	34.60
62	350.00	190.00	150.00	876.00	830.00	1.82	28.00	30.50
63	437.00	176.00	80.00	924.00	743.00	0.90	180.00	83.20
64	100.00	180.00	80.00	876.00	751.00	0.70	56.00	12.00
65	247.00	185.40	165.00	846.00	845.00	0.12	7.00	21.20
66	260.00	227.90	170.00	837.00	717.00	0.70	28.00	27.80
67	250.00	281.05	261.00	837.00	478.00	0.50	7.00	9.60
68	300.00	190.00	200.00	735.00	910.00	1.35	28.00	45.26
69	180.00	166.50	270.00	850.00	850.00	0.21	7.00	14.70
70	218.00	178.00	373.00	700.00	743.00	0.51	182.00	49.60
71	250.00	225.50	160.00	837.00	746.00	1.00	28.00	26.70
72	382.50	189.00	67.50	612.00	1109.00	4.60	7.00	13.10

Sample	C (kg/m^3)	W (kg/m^3)	Class F FA (kg/m3)	CA (kg/m3)	FA (kg/m3)	SP (%)	Age (days)	f'c (MPa)
73	300.00	175.00	200.00	663.00	923.00	1.50	3.00	48.51
74	248.00	175.90	203.00	900.00	808.00	0.35	28.00	50.00
75	405.00	189.00	45.00	612.00	1109.00	4.60	7.00	12.77
76	197.00	137.90	197.00	856.00	856.00	0.28	7.00	22.90
77	218.00	178.00	373.00	700.00	743.00	0.51	90.00	48.90
78	322.00	161.00	138.00	1058.20	693.81	0.24	3.00	32.77
79	198.00	146.20	232.00	900.00	874.00	0.20	28.00	46.00
80	386.00	193.00	72.00	1190.00	523.00	0.42	56.00	48.80
81	355.00	164.00	143.00	1064.00	581.00	0.63	90.00	44.52
82	200.00	160.00	200.00	843.00	842.00	0.17	28.00	34.90
83	180.00	166.50	270.00	850.00	850.00	0.21	28.00	23.65
84	198.00	154.80	232.00	900.00	872.00	0.50	28.00	52.00
85	183.00	188.65	160.00	837.00	891.00	0.50	90.00	34.20
86	197.00	137.90	197.00	856.00	856.00	0.28	28.00	38.90
87	238.00	158.80	159.00	844.00	844.00	0.29	1.00	10.70
88	300.00	200.00	200.00	860.00	845.00	1.80	28.00	26.10
89	440.00	225.50	110.00	590.00	910.00	2.00	365.00	54.60
90	503.00	183.00	158.00	700.00	735.00	0.76	7.00	58.80
91	276.00	161.00	184.00	1058.20	693.81	0.24	3.00	26.50
92	300.00	175.00	200.00	663.00	923.00	1.50	28.00	54.61
93	230.00	192.40	140.00	837.00	864.00	0.80	7.00	19.30
94	290.00	180.00	318.00	700.00	741.00	0.66	7.00	34.40
95	290.00	175.50	100.00	837.00	913.00	0.80	28.00	42.70
96	115.00	167.00	336.00	924.00	743.00	0.65	180.00	37.20
97	333.00	173.00	162.00	924.00	743.00	0.75	28.00	58.50
98	350.00	214.00	170.00	792.00	774.00	0.81	90.00	61.10
99	250.00	295.20	160.00	837.00	566.00	0.50	28.00	11.00
100	386.00	193.00	161.00	1190.00	434.00	0.35	28.00	44.70
101	163.00	163.20	245.00	851.00	851.00	0.20	28.00	26.20
102	170.00	159.10	200.00	900.00	928.00	0.50	1.00	5.00
103	386.00	193.00	72.00	1190.00	523.00	0.42	7.00	29.20
104	115.00	167.00	336.00	924.00	743.00	0.65	28.00	16.00
105	220.00	156.00	180.00	900.00	916.00	0.35	1.00	12.50
106	225.00	180.00	225.00	818.00	818.00	0.35	90.00	45.93
107	248.00	176.00	203.00	900.00	808.00	0.35	1.00	12.00
108	250.00	225.50	160.00	837.00	742.00	0.50	90.00	39.20
109	210.00	193.50	220.00	837.00	768.00	0.80	90.00	45.60
110	325.00	195.00	109.00	896.00	747.00	0.75	90.00	32.41
111	465.00	225.50	85.00	590.00	910.00	1.95	90.00	59.04
112	135.00	180.00	315.00	805.00	805.00	0.26	28.00	36.09
113	322.00	161.00	138.00	1058.20	693.81	0.24	28.00	57.34
114	220.00	176.00	330.00	881.00	686.00	1.21	28.00	47.50
115	290.00	180.00	318.00	700.00	741.00	0.66	182.00	59.90
116	440.00	231.00	110.00	612.00	909.00	3.81	7.00	17.57
117	330.00	176.00	220.00	899.00	700.00	1.35	28.00	60.90
118	360.00	189.00	90.00	612.00	1109.00	4.60	28.00	21.66

Sample	C (kg/m^3)	W (kg/m^3)	Class F FA (kg/m3)	CA (kg/m3)	FA (kg/m3)	SP (%)	Age (days)	f'c (MPa)
119	412.50	231.00	137.50	612.00	909.00	3.81	7.00	18.68
120	386.00	193.00	161.00	1190.00	434.00	0.35	119.00	54.50
121	412.50	231.00	137.50	612.00	909.00	3.81	28.00	25.12
122	300.00	180.00	40.00	876.00	813.00	0.70	7.00	43.50
123	250.00	225.50	160.00	837.00	746.00	1.00	7.00	15.80
124	232.00	135.45	155.00	847.00	846.00	0.38	1.00	16.60
125	270.00	247.50	180.00	837.00	647.00	0.60	28.00	24.50
126	415.00	231.00	135.00	590.00	910.00	1.80	28.00	31.50
127	467.50	231.00	82.50	612.00	909.00	3.81	28.00	18.30
128	250.00	281.05	261.00	837.00	478.00	0.50	28.00	17.00
129	270.00	198.00	180.00	842.00	801.00	0.66	28.00	44.70
130	225.00	170.00	247.00	924.00	743.00	0.68	90.00	49.30
131	232.00	135.45	155.00	847.00	846.00	0.38	7.00	31.30
132	325.00	190.00	175.00	735.00	910.00	1.35	28.00	42.56
133	350.00	302.08	162.00	840.00	768.00	0.09	28.00	51.70
134	495.00	231.00	55.00	612.00	909.00	3.81	28.00	17.20
135	380.00	184.00	145.00	854.00	788.00	0.42	7.00	53.20
136	200.00	160.00	200.00	843.00	842.00	0.17	7.00	19.30
137	333.00	173.00	162.00	924.00	743.00	0.75	180.00	68.00
138	276.00	161.00	184.00	1058.20	693.81	0.24	90.00	71.66
139	260.00	215.00	260.00	775.00	757.00	0.77	90.00	39.30
140	350.00	190.00	150.00	735.00	910.00	1.35	28.00	45.11
141	170.00	159.10	200.00	900.00	930.00	0.20	28.00	31.00
142	180.00	198.00	270.00	829.00	778.00	0.67	28.00	30.30
143	300.00	180.00	40.00	876.00	813.00	0.70	56.00	70.88
144	250.00	225.50	160.00	837.00	742.00	0.50	7.00	13.90
145	450.00	188.00	70.00	844.00	825.00	2.34	365.00	90.60
146	290.00	253.50	100.00	837.00	709.00	0.20	28.00	26.60
147	360.00	198.00	90.00	855.00	813.00	0.71	90.00	68.00
148	230.00	192.40	140.00	837.00	864.00	0.80	28.00	32.10
149	218.00	178.00	373.00	700.00	743.00	0.51	28.00	35.30
150	317.00	262.35	160.00	837.00	594.00	0.50	28.00	29.10
151	163.00	163.20	245.00	851.00	851.00	0.20	7.00	14.70
152	330.00	231.00	220.00	612.00	909.00	3.81	7.00	16.96
153	330.00	176.00	220.00	899.00	700.00	1.35	28.00	60.90
154	200.00	180.00	60.00	876.00	782.00	0.70	56.00	40.44
155	160.00	156.00	240.00	900.00	886.00	0.35	1.00	7.00
156	322.00	161.00	138.00	1058.20	693.81	0.24	7.00	45.76
157	350.00	178.00	186.00	851.00	786.00	0.45	1.00	18.30
158	386.00	193.00	161.00	1190.00	434.00	0.35	7.00	26.10
159	437.00	176.00	80.00	924.00	743.00	0.90	90.00	78.00
160	415.00	231.00	135.00	590.00	910.00	1.80	90.00	43.87
161	238.00	158.80	159.00	844.00	844.00	0.29	7.00	25.80
162	220.00	156.00	180.00	900.00	916.00	0.60	28.00	43.00
163	350.00	235.11	111.00	900.00	831.00	0.15	28.00	61.00
164	330.00	231.00	220.00	612.00	909.00	3.81	28.00	19.93

Sample	C (kg/m^3)	W (kg/m^3)	Class F FA (kg/m3)	CA (kg/m3)	FA (kg/m3)	SP (%)	Age (days)	f'c (MPa)
165	503.00	183.00	158.00	700.00	735.00	0.76	90.00	71.70
166	218.00	178.00	373.00	700.00	743.00	0.51	7.00	21.60
167	355.00	242.00	195.00	590.00	910.00	1.80	365.00	43.70
168	225.00	189.00	225.00	612.00	1109.00	4.60	28.00	19.21
169	280.00	156.00	120.00	900.00	946.00	0.35	1.00	16.00
170	350.00	178.00	186.00	851.00	786.00	0.45	7.00	51.10
171	210.00	193.50	220.00	837.00	768.00	0.80	28.00	26.70
172	300.00	180.00	40.00	876.00	813.00	0.70	1.00	16.66
173	350.00	211.20	90.00	923.00	852.00	0.14	28.00	46.50
174	348.00	286.00	224.00	848.00	783.00	0.43	28.00	58.60
175	375.00	195.00	125.00	735.00	910.00	1.35	28.00	49.39
176	315.00	180.00	135.00	831.00	831.00	0.40	90.00	50.13
177	197.00	137.90	197.00	856.00	856.00	0.28	1.00	7.80
178	380.00	252.00	145.00	659.00	988.00	0.13	28.00	65.50
179	325.00	333.75	120.00	850.00	755.00	0.43	28.00	32.20
180	220.00	156.00	180.00	900.00	916.00	0.60	1.00	12.00
181	247.00	185.40	165.00	846.00	845.00	0.12	1.00	8.70
182	290.00	229.50	220.00	837.00	625.00	0.20	7.00	11.50
183	250.00	225.50	160.00	837.00	739.00	0.00	28.00	27.30
184	467.50	231.00	82.50	612.00	909.00	3.81	7.00	16.71
185	350.00	273.36	186.00	851.00	786.00	0.11	28.00	70.40
186	375.00	175.00	125.00	679.00	938.00	1.50	7.00	59.90
187	330.00	176.00	220.00	899.00	700.00	1.35	90.00	77.90
188	375.00	175.00	125.00	679.00	938.00	1.50	28.00	61.33
189	380.00	200.00	192.00	621.00	931.00	0.43	28.00	67.80
190	183.00	188.65	160.00	837.00	891.00	0.50	7.00	12.00
191	100.00	180.00	80.00	876.00	751.00	0.70	7.00	5.77
192	180.00	198.00	270.00	829.00	778.00	0.67	90.00	42.50
193	380.00	297.86	192.00	621.00	931.00	0.10	28.00	67.80
194	225.00	175.00	275.00	652.00	908.00	1.50	3.00	40.23
195	270.00	247.50	180.00	837.00	647.00	0.60	90.00	37.20
196	180.00	166.50	270.00	850.00	850.00	0.21	90.00	32.32
197	250.00	210.00	250.00	856.00	856.00	1.72	28.00	21.50
198	380.00	184.00	145.00	854.00	788.00	0.42	1.00	26.90
199	270.00	189.00	180.00	612.00	1109.00	4.60	7.00	17.56
200	240.00	202.80	150.00	837.00	831.00	0.60	7.00	19.00
201	250.00	295.20	160.00	837.00	566.00	0.50	90.00	17.20
202	250.00	225.50	160.00	837.00	739.00	0.00	7.00	15.50
203	225.00	175.00	275.00	652.00	908.00	1.50	28.00	41.95
204	250.00	295.20	160.00	837.00	566.00	0.50	7.00	6.20
205	386.00	193.00	72.00	1190.00	523.00	0.42	28.00	35.30
206	225.00	170.00	247.00	924.00	743.00	0.68	28.00	37.20
207	368.00	161.00	92.00	1058.20	693.81	0.24	7.00	52.49
208	220.00	156.00	180.00	900.00	916.00	0.35	28.00	46.00
209	225.00	170.00	247.00	924.00	743.00	0.68	180.00	56.40
210	300.00	190.00	200.00	735.00	910.00	1.35	3.00	20.09

Sample	C (kg/m^3)	W (kg/m^3)	Class F FA (kg/m3)	CA (kg/m3)	FA (kg/m3)	SP (%)	Age (days)	f'c (MPa)
211	292.50	189.00	157.50	612.00	1109.00	4.60	7.00	18.44
212	250.00	155.80	160.00	837.00	919.00	0.50	28.00	36.30
213	290.00	229.50	220.00	837.00	625.00	0.20	28.00	32.90
214	163.00	163.20	245.00	851.00	851.00	0.20	1.00	4.90
215	440.00	176.00	110.00	917.00	714.00	1.35	28.00	69.80
216	238.00	158.80	159.00	844.00	844.00	0.29	28.00	37.80
217	250.00	225.50	160.00	837.00	742.00	0.50	90.00	40.96
218	100.00	180.00	80.00	876.00	751.00	0.70	28.00	10.66
219	290.00	253.50	100.00	837.00	709.00	0.20	7.00	12.80
220	400.00	180.00	20.00	876.00	845.00	0.70	1.00	18.88
221	250.00	225.50	160.00	837.00	742.00	0.50	7.00	14.70
222	327.00	265.00	173.00	803.00	902.00	0.20	28.00	61.60
223	225.00	180.00	225.00	818.00	818.00	0.35	28.00	42.67
224	386.00	193.00	72.00	1190.00	523.00	0.42	119.00	51.70
225	240.00	202.80	150.00	837.00	831.00	0.60	28.00	30.90
226	290.00	175.50	100.00	837.00	913.00	0.80	7.00	32.30
227	386.00	193.00	72.00	1190.00	523.00	0.42	3.00	23.30
228	207.00	186.30	207.00	843.00	845.00	0.40	28.00	33.20
229	368.00	161.00	92.00	1058.20	693.81	0.24	90.00	74.61
230	465.00	225.50	85.00	590.00	910.00	1.95	365.00	61.40
231	250.00	225.50	160.00	837.00	742.00	0.50	28.00	25.30
232	250.00	225.50	160.00	837.00	742.00	0.50	28.00	24.10
233	315.00	180.00	135.00	831.00	831.00	0.40	28.00	38.28
234	276.00	161.00	184.00	1058.20	693.81	0.24	28.00	54.99
235	440.00	225.50	110.00	590.00	910.00	2.00	28.00	33.31
236	385.00	236.50	165.00	590.00	910.00	1.80	90.00	42.07
237	270.00	198.00	180.00	842.00	801.00	0.66	90.00	60.30
238	380.00	252.00	145.00	854.00	788.00	0.10	28.00	73.50
239	275.00	231.00	275.00	612.00	909.00	3.81	7.00	12.32
240	368.00	161.00	92.00	1058.20	693.81	0.24	3.00	39.65
241	380.00	184.00	145.00	854.00	788.00	0.42	28.00	73.50
242	280.00	258.50	190.00	837.00	599.00	0.70	28.00	24.00
243	210.00	201.50	100.00	837.00	910.00	0.80	90.00	28.00
244	495.00	231.00	55.00	612.00	909.00	3.81	7.00	16.51
245	315.00	189.00	135.00	612.00	1109.00	4.60	7.00	25.33
246	135.00	180.00	315.00	805.00	805.00	0.26	90.00	39.88
247	440.00	176.00	110.00	917.00	714.00	1.35	90.00	84.40
248	280.00	156.00	120.00	900.00	946.00	0.35	28.00	45.00
249	200.00	180.00	60.00	876.00	782.00	0.70	28.00	33.33
250	350.00	190.00	150.00	876.00	830.00	1.82	90.00	39.55
251	220.00	180.00	180.00	900.00	850.00	0.35	28.00	38.00
252	61.00	140.70	241.00	864.00	866.00	0.30	1.00	7.30
253	437.00	176.00	80.00	924.00	743.00	0.90	28.00	69.70
254	390.00	195.00	156.00	844.00	779.00	0.71	28.00	53.00
255	450.00	188.00	70.00	844.00	825.00	2.34	28.00	70.80
256	250.00	225.50	160.00	837.00	742.00	0.50	7.00	16.56

Sample	C (kg/m^3)	W (kg/m^3)	Class F FA (kg/m3)	CA (kg/m3)	FA (kg/m3)	SP (%)	Age (days)	f'c (MPa)
257	386.00	193.00	72.00	1190.00	523.00	0.42	7.00	24.50
258	325.00	190.00	175.00	735.00	910.00	1.35	7.00	29.79
259	210.00	279.50	220.00	837.00	562.00	0.20	7.00	6.20
260	355.00	242.00	195.00	590.00	910.00	1.80	28.00	29.71
261	385.00	231.00	165.00	612.00	909.00	3.81	7.00	26.71
262	237.00	159.10	133.00	900.00	960.00	0.50	1.00	16.00

Test dataset

Sample	C (kg/m^3)	W (kg/m^3)	Class F FA (kg/m3)	CA (kg/m3)	FA (kg/m3)	SP (%)	Age (days)	f'c (MPa)
263	285.50	188.10	285.00	788.70	788.70	0.40	28.00	58.75
264	386.00	193.00	72.00	1190.00	523.00	0.42	28.00	42.30
265	385.00	236.50	165.00	590.00	910.00	1.80	365.00	44.90
266	503.00	183.00	158.00	700.00	735.00	0.76	28.00	68.40
267	350.00	178.00	186.00	851.00	786.00	0.45	28.00	70.40
268	300.00	190.00	200.00	735.00	910.00	1.35	130.00	59.04
269	315.00	189.00	135.00	612.00	1109.00	4.60	28.00	28.95
270	290.00	175.50	100.00	837.00	913.00	0.80	90.00	55.90
271	385.00	231.00	165.00	612.00	909.00	3.81	28.00	30.01
272	169.00	190.35	254.00	853.00	853.00	0.00	7.00	15.60
273	386.00	193.00	161.00	1190.00	434.00	0.35	3.00	24.80
274	385.00	236.50	165.00	590.00	910.00	1.80	28.00	30.70
275	427.00	243.90	115.00	844.00	779.00	0.12	28.00	59.40
276	386.00	193.00	161.00	1190.00	434.00	0.35	7.00	31.30
277	503.00	183.00	158.00	700.00	735.00	0.76	182.00	69.50
278	210.00	201.50	100.00	837.00	910.00	0.80	7.00	11.10
279	350.00	190.00	150.00	735.00	910.00	1.35	3.00	24.11
280	300.00	175.00	200.00	663.00	923.00	1.50	7.00	48.98
281	290.00	180.00	318.00	700.00	741.00	0.66	90.00	62.50
282	169.00	190.35	254.00	853.00	853.00	0.00	28.00	30.20
283	260.00	215.00	260.00	775.00	757.00	0.77	365.00	54.90
284	368.00	161.00	92.00	1058.20	693.81	0.24	28.00	64.96
285	290.00	180.00	318.00	700.00	741.00	0.66	28.00	54.00
286	210.00	201.50	100.00	837.00	910.00	0.80	28.00	19.10
287	250.00	210.00	250.00	856.00	856.00	1.72	90.00	30.32
288	220.00	132.00	180.00	900.00	982.00	0.35	28.00	51.00
289	386.00	193.00	72.00	1190.00	523.00	0.42	90.00	50.50
290	115.00	167.00	336.00	924.00	743.00	0.65	7.00	8.40
291	375.00	195.00	125.00	735.00	910.00	1.35	3.00	27.66
292	210.00	279.50	220.00	837.00	562.00	0.20	28.00	10.20
293	200.00	160.00	200.00	843.00	842.00	0.17	1.00	7.00
294	135.00	180.00	315.00	805.00	805.00	0.26	7.00	17.47
295	61.00	140.70	241.00	864.00	866.00	0.30	28.00	35.80
296	200.00	180.00	60.00	876.00	782.00	0.70	7.00	21.77
297	115.00	167.00	336.00	924.00	743.00	0.65	90.00	26.10

Sample	C (kg/m^3)	W (kg/m^3)	Class F FA (kg/m3)	CA (kg/m3)	FA (kg/m3)	SP (%)	Age (days)	f'c (MPa)
298	290.00	253.50	100.00	837.00	709.00	0.20	90.00	35.70
299	315.00	180.00	135.00	831.00	831.00	0.40	7.00	32.07
300	220.00	176.00	330.00	881.00	686.00	1.21	28.00	47.50
301	61.00	140.70	241.00	864.00	866.00	0.30	7.00	20.60
302	317.00	262.35	160.00	837.00	594.00	0.50	7.00	17.00
303	220.00	156.00	180.00	900.00	916.00	0.35	1.00	13.00
304	337.50	189.00	112.50	612.00	1109.00	4.60	28.00	22.71
305	275.00	231.00	275.00	612.00	909.00	3.81	28.00	16.90
306	400.00	180.00	20.00	876.00	845.00	0.70	28.00	55.77
307	386.00	193.00	161.00	1190.00	434.00	0.35	56.00	51.20
308	360.00	198.00	90.00	855.00	813.00	0.71	28.00	52.10
309	386.00	193.00	161.00	1190.00	434.00	0.35	28.00	36.90
310	210.00	279.50	220.00	837.00	562.00	0.20	90.00	19.70
311	276.00	161.00	184.00	1058.20	693.81	0.24	7.00	43.76
312	427.50	188.10	142.50	812.10	812.10	0.50	28.00	50.00
313	300.00	180.00	40.00	876.00	813.00	0.70	28.00	58.88
314	322.00	161.00	138.00	1058.20	693.81	0.24	90.00	72.61
315	169.00	190.35	254.00	853.00	853.00	0.00	1.00	5.20
316	382.50	189.00	67.50	612.00	1109.00	4.60	28.00	16.56
317	350.00	190.00	150.00	735.00	910.00	1.35	130.00	55.24
318	440.00	231.00	110.00	612.00	909.00	3.81	28.00	23.27
319	210.00	193.50	220.00	837.00	786.00	0.80	7.00	15.50
320	386.00	193.00	161.00	1190.00	434.00	0.35	90.00	53.80
321	207.00	186.30	207.00	843.00	845.00	0.40	7.00	17.40
322	270.00	247.50	180.00	837.00	647.00	0.60	7.00	14.60
323	465.00	225.50	85.00	590.00	910.00	1.95	28.00	35.33
324	170.00	157.00	200.00	900.00	930.00	0.20	1.00	6.00
325	220.00	156.00	180.00	900.00	916.00	0.35	28.00	49.00
440.00	225.50	110.00	590.00	910.00	2.00	90.00	52.80	440.00
200.00	180.00	60.00	876.00	782.00	0.70	1.00	4.44	200.00
